# Case-Based Questions for Teaching Emergency Medicine Pharmacotherapy

**DOI:** 10.21980/J8PW61

**Published:** 2021-01-15

**Authors:** David Eichenberger, Gary Pollock, Luke Huber, Aaron Brown, David Zimmerman

**Affiliations:** * University of Pittsburgh Medical Center, Department of Emergency Medicine, Pittsburgh, PA; ^ Duquesne University, Division of Pharmacy Practice, Pittsburgh, PA

## Abstract

**Audience:**

This pharmacotherapy curriculum is designed for emergency medicine residents of all postgraduate years and could also be given to rotating medical students during clerkships.

**Length of Curriculum:**

Curriculum is to run monthly for an 18-month general curriculum cycle

**Introduction:**

Pharmacotherapy is a critical part of day-to-day practice of Emergency Medicine (EM). The purpose of this innovation is to give this subject specific dedicated instruction time. We introduced a dedicated pharmacotherapy curriculum as part of our conference time through a series of case-based question sets that mirrored our educational blocks.

**Educational Goals:**

Our goals were to teach residents clinical applications of EM pharmacotherapy including drug selection and consideration of alternatives, interactions, and adverse effects, as well as to prepare them for pharmacotherapy questions on board examinations.

**Educational Methods:**

The educational strategies used in this curriculum include: case-based vignettes, multiple choice assessments, and guided review explanation and discussion. Questions and explanations are written by resident physicians using a variety of textbooks and online resources and are then reviewed, edited, and expanded upon by attending physicians and an EM pharmacist.

**Research Methods:**

This curriculum was implemented in the University of Pittsburgh Emergency Medicine residency program. Curriculum is ongoing and initial data covers a 4-month pilot period. Survey questionnaires were given before and after, using the 7-point Likert scale (1 strongly agree to 7 strongly disagree) for self-assessed knowledge and satisfaction with the curriculum. Primary measure was resident agreement with the statement, “I am confident in overall knowledge of EM pharmacotherapy.” We also surveyed readiness for independent practice, knowledge appropriate for training level, board exam preparedness, and satisfaction with curriculum.

**Results:**

On the whole residents did feel their pharmacology knowledge improved. Our primary marker was response to a survey question, “I am confident in my overall knowledge of EM pharmacotherapy.” In our sample of 30 residents, this question received a pretest score of 3.7 on a 7-point Likert scale (1 strongly agree, 7 strongly disagree). On post intervention surveys this score had improved to 2.6 (p = .00008). In general, residents appreciated this change in curriculum structure. Resident assessment of their improvement during the curriculum was 2.1, aligning with “agree.” Satisfaction also improved from a 3.8 on initial survey to a 3.1, with statistical significance (p =.023).

**Discussion:**

We had success in our primary outcome of self-assessed resident learning as above. Satisfaction also improved. Residents assessed self-improvement in knowledge relative to year of training, clinical practice ability, and independent practice ability by statistically significant amounts. In addition, the assessments provided the residency clinical competency committee with objective knowledge of pharmacotherapy-based topics. We feel this easy to implement and effective curriculum would be generalizable across programs looking to add options for teaching EM pharmacotherapy, or to other programs looking to add a formal instruction and evaluation component to traditionally informal curriculum.

**Topics:**

Emergency Medicine, education, pharmacotherapy, case based, questions, milestones.

## USER GUIDE

**Table:** 

List of Resources:	
Abstract	35
User Guide	37
Curriculum Chart	41
Learner Materials	42
Cardiology Block Questions	42
ENT and Ophthalmology Block Questions	47
Gastroenterology Block Questions	52
Hematology and Oncology Block Questions	56
Infectious Disease Block Questions	60
Musculoskeletal Block Questions	64
Pulmonary and Critical Care Block Questions	69
Trauma Block Questions	75
Instructor Materials	76
Cardiology Block Answers	76
ENT and Ophthalmology Block Answers	85
Gastroenterology Block Answers	92
Hematology and Oncology Block Answers	98
Infectious Disease Block Answers	103
Musculoskeletal Block Answers	109
Pulmonary and Critical Care Block Answers	116
Trauma Block Answers	124

**Learner Audience:**

Medical Students, Interns, Junior Residents, Senior Residents

**Length of Curriculum:**

The curriculum design targets 18 months, which is the length of our curriculum cycle, could be condensed or elongated to 12 or 24 months based on institutional needs. Our efficacy data is based on a 4-month pilot program at the inception of this project.

**Topics:**

Emergency Medicine, education, pharmacotherapy, case based, questions, milestones.

**Objectives:**

The learner should seek to improve knowledge of appropriate pharmacotherapy treatments for common and life-threatening emergency medicine presentations, as well as common and clinically significant contraindications and side effects. Knowledge improvements are to be assessed pre- and post-completion of the curriculum.

The specific objectives modeled after the ABEM milestones: PT2, PT5, PT6, PC5 level 3 and 4, PC 11, PC 13 and are as follows:

1. Select appropriate pharmaceutical for both rare and common emergency medicine presentations
2. Identify relative and absolute contraindications to specific pharmacotherapy
3. Identify alternative therapies for patient allergy considerations
4. Identify side effects and other effects of pharmacotherapy

### Brief introduction

Knowledge of pharmacotherapy is essential in the day-to-day practice of the emergency medicine physician. According to the CDC, an estimated 368.5 million drugs are dispensed in emergency departments annually, encompassing 81.1% of ED patient visits.^1^ In our program, a pharmacology curriculum was not previously formally codified and was largely taught in real-time, on shift, or incorporated into lectures on other topics. We sought to create a more formalized process. A case-based fashion with multiple-choice questions was chosen to mirror the in-service examination and emergency medicine boards, as well as a platform for discussion of specific pharmacological topics. Multiple-choice questions have been studied and validated as a means of general instruction, as well as improving recall and improving performance on subsequent examinations.^2^,^3^ Feedback following questions has been shown to improve long term retention of information.^4^ These principles have been applied to many other areas of medical education with improvement in knowledge retention such as infectious disease fellowship curriculum.^5^ We sought to apply these instructional methods to the topic of EM pharmacotherapy.

### Problem identification, general and targeted needs assessment

The American Board of Emergency Medicine (ABEM) and the Accreditation Council for Graduate Medical Education (ACGME) have published milestones for the competency-based assessment of EM residents which represent “a matrix of the knowledge, skills, abilities, attitudes, and experiences that should be acquired during specialty training in Emergency Medicine.”^6^ We are specifically addressing the PC 5 milestone:

Selects and prescribes appropriate pharmaceutical agents based upon relevant considerations such as mechanism of action, intended effect, financial considerations, possible adverse effects, patient preferences, allergies, potential drug-food and drug-drug interactions, institutional policies, and clinical guidelines, and effectively combines agents and monitors and intervenes in the advent of adverse effects in the ED.

The purpose of this innovation was to address a deficiency in the current didactic curriculum design by creating a dedicated curriculum and assessment of the pharmacotherapy milestone. In our institution, the pharmacotherapy content was taught through occasional lecture points or as needed on shift, but without any formal curriculum. Unlike other milestones that have dedicated rotations, such as ultrasound or prehospital care, or more rigorously codified expectations and evaluations, i.e., procedures (such as vascular access) and medical knowledge (in-service scores), pharmacotherapy was more difficult to assess and standardize teaching. Our Clinical Competence Committee repeatedly had little subjective or objective evidence regarding this milestone leading to anecdote-based assessment or general gestalt among the committee. Adding an entire pharmacy rotation is impractical, but a more focused and longitudinal educational initiative could be undertaken. Additionally, this curriculum allows for more direct assessment of this milestone that was not previously directly addressed at this institution.^7^

Previously published curricula include those for instructing an entire organ system block or one focused area of instruction. We did not find a published curriculum of one milestone instructed in a longitudinal fashion across organ system/topic blocks. We sought to provide a formal and specifically directed curriculum in parallel with our longitudinal 18-month curriculum. This means of instruction draws inspiration from national question banks used in preparation for board examinations such as USMLE and ABEM board certification. It has been shown that completion of more unique USMLE prep questions was correlated with improved score.^8^ We sought to apply this approach to the more focused subject matter of EM pharmacotherapy. We sought to combine clinical teaching pearls, board content, and topics from the ABEM model of clinical practice using case-based situations. The framework included approximately 10 questions during each organ system block. The curriculum had the potential to impact all residents by providing a formal source of pharmacotherapy instruction. The confidence of our residents in pharmacotherapy topics was assessed prior to the initiation of the curriculum and scores suggested an educational need for an additional focus on pharmacotherapy.

### Goals of the curriculum

The goal of this curriculum is to educate EM residents of all PGY years in the clinical practice of EM pharmacotherapy and prepare them for in-service and board examination. They should be able to correctly determine the indicated pharmacotherapy for a given condition and consider alternatives, contraindications, and adverse effects. The curriculum also serves to give residency clinical competency committee a means for assessing progress along the pharmacotherapy milestone.

### Objectives of the curriculum

The learner should seek to improve knowledge of appropriate pharmacotherapy treatments for common and life-threatening emergency medicine presentations, as well as common and clinically significant contraindications and side effects.

Knowledge improvements are to be assessed pre- and post-completion of the curriculum.

The specific objectives modeled after the ABEM milestones: PT2, PT5, PT6, PC5 level 3 and 4, PC 11, PC 13 and are as follows:

1. Select appropriate pharmaceutical for both rare and common emergency medicine presentations
2. Identify relative and absolute contraindications to specific pharmacotherapy
3. Identify alternative therapies for patient allergy considerations
4. Identify side effects and other effects of pharmacotherapy

### Educational strategies

Educational strategies used include clinical vignettes followed by multiple choice questions to elicit knowledge, application, and recall of EM pharmacotherapy. These question sets were followed by a period of review and discussion to further solidify this knowledge because long term retention is improved by a period of feedback following multiple choice testing.^9^ Residents were given 10–15 minutes to complete a paper copy of the question set. Immediately following the completion of the question set by residents, the questions were then discussed as a large group. Discussion was led by the resident responsible for writing the questions along with faculty. Approximately 1–2 minutes were spent on each question discussing the reasoning and pertinent pharmacology behind the correct answers as well as the incorrect answers. If a resident was unable to attend educational conference in person, an electronic form of the quiz was sent via e-mail. Once the absent resident returned a completed version of the quiz, they were then sent an answer key with explanations of the correct and incorrect answers. This educational curriculum is focused on the core content of emergency medicine and is foundational knowledge to the specialty. It is relevant and appropriate for learners of all residency years as well as medical students.

### Results and tips for successful implementation

We implemented this during our regular educational conference as opposed to adding an additional asynchronous requirement. We felt this was useful and well-received as a means to provide additional education and instruction without placing additional time burdens on busy residents. Other options could include asynchronous completion of the questions with in-person review. We collected survey data pre- and post-pilot period implementation of this curriculum after 4 months. We provided the curriculum to residents of all three PGY years for a total of 48 residents at a time. Given a transition of academic years during the study period, we had a total of 54 residents and some additional medical students who participated in this curriculum. We collected completed efficacy data on 30 of the 32 residents completing the pre-intervention and post-intervention surveys. We chose this population because they were the two classes of residents who would be in training throughout the entire study period, thereby allowing us to perform matched analysis of the pre- and post-intervention scores. We measured pre- and post-test self-assessed knowledge and satisfaction with the curriculum after the first 4 months of implementation using a survey with several questions using a 7-point Likert scale from 1 (strongly agree) to 7 (strongly disagree).

For our statistical analysis, we used a Wilcoxon signed rank test, given our sample of paired data from the 30 residents that completed both surveys. Our primary marker was the response to, “I am confident in my overall knowledge of EM pharmacotherapy.” This received a pre-intervention score of 3.7 and a post score of 2.6 (*p* = 0.00008), achieving statistical significance. For the survey item, “I am confident in my ability to apply my knowledge of EM pharmacotherapy to clinical practice,” we saw an improvement from 3.2 to 2.3 (*p* = 0.0004). Resident assessment of knowledge being appropriate for level of training showed an improvement from a mean of 3.0 to a mean of 2.3 with a (*p =* .010). Our largest degree of improvement was in self-assessed readiness for independent practice, improving from 4.3 to 3.0 (*p* = 0.0003). Satisfaction scores improved from 3.8 to 3.2 (*p =* 0.022), indicating a statistically significant, positively received change from original curriculum style to the intervention.

We hypothesized the potential for improvement in preparedness for the in-service exam. For the residents’ self-assessed level of preparation for the in-service exam, we saw a trend towards a better self-assessed preparation from 3.2 to 2.7, but this did not reach statistical significance (*p* = 0.057). Additional data would need to be gathered to determine any degree of impact on actual results of the exam, and pharmacology related questions are not specifically identified on score reports.

Self-assessed knowledge improvement received a score of 2.2 (SD 0.92). This was only assessed on the post intervention survey, and was a response to item, “My knowledge of EM pharmacotherapy has improved by completing these cases,” corresponding to “agree” on the Likert scale. Participants’ assessment of the efficacy of the intervention, “I believe these cases were an effective means of learning EM pharmacotherapy,” scored a 2.3 (SD 1.1), also corresponding closely to an assessment of agreement.

We also included an area in the feedback form for open response for ways to improve the content and implementation of the curriculum. As a result of this feedback, we are working to expand the explanations of both correct and incorrect answer choices as well as provide additional feedback and discussion time. In the feedback section, one resident raised an interesting point about a possible confounding effect: “If my post-survey does not show improvement in our education following your intervention, it is because your intervention has just made me more aware of how much I don’t know.” The very act of education can raise awareness of knowledge gaps that were not reflected in the pretest surveys.

It is possible our finding of such improvement in independent practice readiness could be confounded by the time that had passed during the instruction period, moving all residents closer to the time they would be expected to possess independent practice capabilities. However, we would expect at least a substantial portion of this change arises from our targeted intervention, and we limited the time from the before and after survey to limit changes due external knowledge and forces.

**Table:** 

Mean Likert Scale rating		
Survey Item	Pre-Intervention	Post-Intervention
I am confident in my ability to apply my knowledge of EM pharmacotherapy to clinical practice.	3.1	2.2
I am confident in my overall knowledge of EM pharmacotherapy.	3.6	2.6
I possess an adequate level of knowledge of EM pharmacotherapy to independently practice EM.	4.1	2.9
My knowledge of EM pharmacotherapy has improved by completing these cases.	Not Applicable	2.1

**Table:** 

Demographics	

Sex	

Male	Female
24(80%)	6 (20%)

PGY at Start of Pilot Period	

PGY-1	PGY2
14 (47%)	16 (53%)

### Associated content

Please see attached Questions and Answer Blocks.

### Evaluation and feedback

In addition to our expanded explanations detailed above, we are working on creating an expanded question bank to allow for ongoing application because our program education cycle repeats every 1.5 years. As the question bank is developed, there will be an increasingly large bank from which to pull questions related to a specific subject matter. For residents unable to attend educational conferences due to clinical or other obligations, a copy of the quiz was sent via email and an answer key with explanations of the correct and incorrect answers was sent once they returned the completed quiz. Constructive feedback mostly centered on increasing extent and quality of explanations for incorrect answers, which have been added to the attached materials. We did find a previous study demonstrating a correlation between resident performance on regular multiple-choice quizzes and the in-service examination, and it may be possible in further study to correlate our quiz scores with in-service examinations, or possibly as a means to identify a lower-performing cohort of residents for early intervention or extra instruction.^10^

## Figures and Tables

**Table:** 

Topic	Recommended Educational Strategy	Educational Content	Objectives	Learners	Timing, Resources Needed	Recommended Assessment, Milestones Addressed
Pharmacotherapy	To follow along with general curriculum, ie, quizzes follow regularly scheduled educational content for a given organ system or topic Quizzes during or just after educational block with discussion following	- series of case-based vignettes with multiple choice questions - answers and explanations provided - learner questions and learner focused discussion to follow	The learner will demonstrate ability to: Select appropriate pharmaceutical for both rare and common emergency medicine presentations Identify relative and absolute contraindications to specific pharmacotherapy Identify alternative therapies for patient with allergy considerations Identify side effects and other effects of pharmacotherapy	All resident PGY years as well as any rotating medical students	15–20 minutes per question set for completion, and review/discussion can be rearranged to fit program specific needs 30 minutes of administrative time for scoring and tracking	Milestone:PT2, PT5, PT6, PC5 level 3 and 4, PC 1, PC 13 Assessment: Quiz

## LEARNER MATERIALS

### Cardiology Block Questions

1. A 65-year-old man with a history of hypertension, atrial fibrillation, CHF (congestive heart failure), and depression presents to the emergency department with altered mental status following an intentional ingestion. His EKG shows bidirectional ventricular tachycardia; which of the following medications did he ingest?
  - Diltiazem
  - Labetalol
  - Fluoxetine
  - Digoxin
2. A 67-year-old man with a history of tertiary syphilis presents to the emergency department with chest pain radiating to his shoulder and R arm weakness. After correctly diagnosing his aortic dissection and establishing adequate rate control and analgesia, he remains hypertensive. You elect to start sodium nitroprusside. What is the mechanism of action of this drug?
  - L type Calcium channel blockade
  - Direct relaxation of smooth muscle
  - Dopamine-1 agonist
  - Release of nitric oxide leading to increased intracellular cGMP (cyclic guanosine monophosphate) formation and smooth muscle relaxation
3. After initiating the initiate treatment in the question above, you remember reading about possible toxicity from this drug in medical school. During an unfortunately long wait for the OR, your patient becomes increasingly altered and develops a lactic acidosis. In addition to stopping the medication, what is the appropriate treatment?
  - Sodium thiosulfate
  - Calcium Chloride
  - D50
  - Glucagon
4. A 33-year-old female presents to the emergency department with 7 days of chest pain and exertion dyspnea. An EKG shows diffuse ST elevations. Which of the following treatments has been shown to have an increased rate of recurrence of her disease process?
  - Aspirin
  - Prednisone
  - Indomethacin
  - Colchicine
5. In which of the following circumstances is procainamide contraindicated for the treatment of wide complex tachycardia?
  - Patient with WPW (Wolff-Parkinson-White) in supraventricular tachycardia
  - Patient successfully cardioverted from ventricular tachycardia
  - Amitriptyline toxicity
  - Stable ventricular tachycardia
6. A 47-year-old man with a history of asthma and atrial fibrillation presents to the emergency department in respiratory distress with wheezing and poor air movement after a treatment with albuterol and ipratropium-bromide administered by medics. You start a continuous albuterol treatment and note the patient to be tachycardic and irregular at a rate of 150, consistent with atrial fibrillation with rapid ventricular response. In addition to fluid resuscitation, what treatment would you initiate for rate control?
  - Metoprolol
  - Verapamil
  - Adenosine
  - Atropine
7. A 3-day-old infant presents to your emergency department by EMS for poor feeding and lethargy. On examination you find a cyanotic infant, with poor tone, tachypnea and central cyanosis. IV access is obtained via a scalp IV. Stat portable CXR shows an “egg on a string” with narrow mediastinum and increased pulmonary vascular markings. What treatment should you empirically initiate?
  - Prostaglandin E at 0.1 mcg/kg/min
  - Prostaglandin E at 5 mcg/kg
  - Prostacyclin at 25 ng/kg/min
  - Prostacyclin at .02 mcg/kg
  - Norepinephrine at .02 mcg/kg/min
8. A 72-year-old female presents to a rural, critical-access hospital complaining of chest pain. She is clutching her chest and diaphoretic. EKG shows ST elevations in lead II, III, aVF. You evaluate her for initiation of fibrinolytic therapy. Which of the following is an absolute contraindication to the use of fibrinolytics?
  - Large ischemic MCA (middle cerebral artery) stroke 4 months ago with residual weakness
  - Breast cancer with metastasis to brain
  - Current use of warfarin
  - SBP (systolic blood pressure) on 210 on arrival to ED
9. A 79-year-old male presents to the emergency department with dyspnea and altered mental status. On arrival vital signs are HR 111 BP 77/50 RR 23 T 37.1°C O2 sat 95% on NRB. Examination reveals 3+ LE edema, 5 second capillary refill with cold extremities, and diffuse rales. You evaluate with bedside echo and see LV (left ventricle) and RV (right ventricle) dilation with EF (ejection fraction) estimated 10–15%. Diagnosing acute decompensated heart failure and cardiogenic shock, you elect to begin inotropic support with milrinone in addition to other therapies. What is the mechanism of action of milrinone?
  - Phosphodiesterase 3 inhibition
  - Beta1 and Beta2 agonism
  - D1–D5 agonism
  - Alpha1, Beta1, Beta2 agonism
10. A 4-year-old female is brought into the emergency department for altered mental status and vomiting. The grandmother’s empty digoxin bottles were found with the child. Which of the following tests is most helpful in establishing a prognosis?
  - Serum potassium
  - EKG
  - Serum calcium
  - Lactate
11. A tall thin 45-year-old man with a history of blindness presents to the emergency department for sudden onset of tearing chest pain radiating to the back. His vital signs are BP 180/130, Pulse 102, RR 23, temp 36.4°C. You note a thoracic aortic dissection on bedside ultrasound at the sternal notch. Which of the following medications should be initiated first?
  - Fenoldopam
  - Esmolol
  - Sodium Nitroprusside
  - Clevidipine
12. A 24-year-old male presents to your emergency department short of breath after just returning from Santiago, Chile. He has rales throughout pulmonary fields and decreased ejection fraction on bedside echo. You suspect Trypanosoma cruzi infection and initiate empiric treatment with?
  - Benznidazole
  - Praziquantel
  - Mefloquine
  - Doxycycline
13. A 19-year-old male presents to the emergency department for chest pain that is worse when lying down at night. You note a small pericardial effusion on bedside echo. Which of the following treatment regimens is associated with fastest resolution of symptoms and lowest rate of recurrence?
  - Prednisone and colchicine
  - Indomethacin and prednisone
  - Colchicine and aspirin
  - Aspirin and indomethacin
14. An 89-year-old woman presents to the emergency department for heart palpitations and dizziness. Her vital signs are HR 220 BP 100/60 RR 18 T 37.2°C O2 sat 96%. She is alert and oriented. The patient is placed on the cardiac monitor which shows a rhythm concerning for ventricular tachycardia. Treatment is initiated with amiodarone. Which of the following is not an acute adverse effect of amiodarone?
  - Hypotension
  - Torsades de pointes
  - AV nodal blockade
  - Pulmonary fibrosis

### ENT and Ophthalmology Block Questions

1. A 64-year-old male presents to the ED complaining of 1.5 days of headache, general malaise, and photophobia. On exam you note a vesicular lesion on the tip of the nose. You perform a slit lamp exam and note pseudodendrites. What is the most important component of treatment for this condition?
  - Oral acyclovir or valacyclovir
  - Erythromycin ointment
  - Prednisolone acetate drops
  - Cyclopentolate drops
2. A 3-year-old male presents to the emergency department with 4 days of fever, irritability, poor oral intake and tugging at his ears. On exam both tympanic membranes are erythematous and bulging. Ocular exam is normal. What is the preferred initial treatment for this condition?
  - Amoxicillin 90mg/kg in two divided doses ×14 days
  - Amoxicillin 45 mg/kg twice per day × 5–7 days
  - Amoxicillin/clavulanate 80mg/kg/day dosed by amox in bid divided dosing ×7–10 days
  - Cefpodoxime 10mg/kg × 7 days
3. If the patient in the above question also had signs of conjunctivitis, what would the preferred treatment be?
  - Azithromycin 10mg/kg per day × 5 days
  - Clindamycin 10 mg/kg TID × 7 days
  - Amoxicillin/clavulanate 45 mg/kg BID dosed by amoxicillin ×7 days
  - Penicillin 25 mg/kg TID ×10 days
4. A 15-year-old female presents to the ED complaining of right ear pain. She is a member of the swim team and is self-conscious of her ear wax and uses q-tips 5 times daily. On exam you note tenderness of the tragus, otorrhea, edema and erythema of the auditory canal and a small perforation of the tympanic membrane. What is the preferred treatment for this condition?
  - Ofloxacin otic drops
  - Ciprofloxacin hydrocortisone drops
  - Neomycin/polymyxin B/hydrocortisone suspension
  - Ciprofloxacin PO
5. If the patient in the above question was a poorly controlled type I diabetic with leukemia on chemotherapy, what treatment would you prescribe?
  - Ofloxacin drops
  - Ciprofloxacin po
  - Clindamycin
  - Cefpodoxime
6. A 17-year-old female with 7 ear piercings, including two high chondral piercings, presents to the ED with severe auricular ear pain, with erythema. What is the treatment of choice?
  - Amoxicillin clavulanate
  - Clindamycin
  - Ciprofloxacin
  - Ceftriaxone
7. A 22-year-old previously healthy, immunized male presents to the ED with right ear pain and swelling, fever and pain in the ear as well as the surrounding area, who has now developed an asymmetric smile on the right side. Examination shows a dull bulging TM (tympanic membrane) as well as an erythematous, boggy, tender mastoid. What is the empirical treatment for this condition?
  - Vancomycin and ceftriaxone
  - Ampicillin
  - Vancomycin and gentamicin
  - Piperacillin-tazobactam
8. A 72-year-old male with a history of CAD (cerebral artery disease) and TIA (transient ischemic attack) on aspirin and Plavix presents with persistent epistaxis. After failing direct pressure and oxymetazoline, you elect to proceed with packing with a pledget soaked in TXA (tranexamic acid). What is the mechanism of action of this agent?
  - Inhibits fibrinolysis by displacing plasminogen from fibrin
  - Alpha 1 receptor agonist causing vasoconstriction
  - Provides an increase in levels of factors II, VII, IX, and X
  - Direct activator of thrombin
9. A 48-year-old female presents to the ED for 5 days of constant facial pain, nasal congestion and pressure, worse with bending over, and subjective fever. On evaluation patient is nontoxic, temp 37.9°C, with tenderness to palpation over maxillary sinuses. Which is the most reasonable treatment for this condition?
  - Amoxicillin-clavulanate
  - Levofloxacin
  - Azithromycin
  - Fluticasone nasal spray and saline irrigation
10. A 22-year-old female presents to your ER with throat pain. Vital signs are HR 109, T 38.6°C, BP 110/72, RR 20. On examination she has tonsillar exudate w/asymmetrical swelling R>L and tender cervical LAD (lymphadenopathy), and two finger trismus. A CT scan shows cellulitis with no discrete fluid collection. Her allergy list includes penicillin (hives), and hydrocodone (vomiting). Which curative therapy would you initiate?
  - Ampicillin- sulbactam
  - Clindamycin
  - Doxycycline
  - Moxifloxacin
11. A 21-year-old male presents to the emergency department 4 days after wisdom tooth extraction with severe pain. Pain had initially subsided before acute worsening. Vital signs are within normal limits including temperature. On examination you see exposed bone with no visible clot in the extraction site. Which of the following should be included in the treatment regimen?
  - Biting down on black tea bags
  - Trimethoprim-sulfamethoxazole
  - Iodoform gauze soaked in clove oil
  - Ampicillin-sulbactam
12. A 47-year-old male, who does not wear contact lenses, presents to the emergency department for the second time in 2 weeks. At his first visit he was found to have an R facial palsy that did not spare the forehead. Today he presents for severe eye pain and decreased visual acuity. Exam reveals a corneal defect w/fluorescein uptake, consensual photophobia, and trace hypopyon. What treatment regimen should be initiated?
  - Erythromycin ointment three times daily
  - Moxifloxacin drops q1 hour
  - Ciprofloxacin drops twice daily
  - Trifluridine drops twice daily
  - Cyclopentolate twice daily
13. A 23-year-old female presents to the ED with right eye pain and redness following a direct blow to the orbit. On examination she has eye pain not relieved by tetracaine. She also has direct and consensual photophobia. Visual acuity 20/100 OD compared to 20/30 OS. Cyclopentolate drops are started as part of her treatment regimen. What is the mechanism of this drug?
  - Sympathomimetic causing cycloplegia and mydriasis
  - Topical anesthetic blocking sodium channels
  - Steroid decreasing inflammation
  - Anticholinergic causing mydriasis and cycloplegia
14. A 48-year-old female with a history of sickle cell disease presents to the emergency department for sudden onset of right-sided eye pain, severe headache, and blurry vision. On evaluation she is noted to have ciliary flush and pupil is fixed and midrange intraocular pressure is 42. Which of the following is contraindicated in the immediate management of this patient?
  - Topical timolol
  - Systemic acetazolamide
  - Topical brimonidine
  - Topical pilocarpine
15. A 54-year-old female with history of poorly controlled diabetes presents to the emergency department with 2 days of sudden onset and rapidly progressive severe nasal, facial, periorbital pain and headache. She has noticed double vision and discoloration of her nose as well. On a physical exam you also notice black discoloration of the palate as well as inability to fully abduct the right eye. In addition to surgical consultation what treatment should you initiate?
  - Fluconazole
  - Amphotericin
  - Vancomycin and piperacillin and tazobactam
  - Clindamycin, ceftriaxone

### Gastroenterology Block Questions

1. A 6-year-old female presents to the emergency department for parental concern over white spots in her mouth. She is well appearing and in no apparent distress. On exam you note white plaques in the oropharynx that scrape off with a tongue depressor. What treatment is most appropriate for this condition?
  - Fluconazole po
  - Liposomal amphotericin swish and spit
  - Chlorhexidine rinses
  - Clotrimazole troche
2. A 28-year-old undomiciled male with HIV presents to the emergency department. He has been unable to fill anti-retroviral prescriptions and is complaining of chest pain, dysphagia, hematemesis and fever. His last CD4 count was 12. On examination patient appears very uncomfortable requiring multiple doses of analgesia. EKG and CXR are within normal limits and on examination no thrush or white plaques are seen. What treatment is most likely to be effective?
  - Ganciclovir
  - Fluconazole
  - Pantoprazole
  - Aspirin
  - Ceftriaxone
3. A 74-year-old man with severe osteoarthritis of the knees presents with epigastric pain and melena. He appears pale, with a heart rate of 104 and hemoglobin of 5.8. Which of the following agents taken daily is most likely responsible for this presentation?
  - Acetaminophen
  - Diclofenac
  - Oxycodone
  - Lavender oil
4. What is the appropriate initial antisecretory therapy for the patient above?
  - Famotidine
  - Pantoprazole
  - Sucralfate
  - Magnesium hydroxide + aluminum hydroxide
  - Octreotide
5. A 37-year-old male with a long-standing history of alcohol abuse presents for massive hematemesis. In addition to airway management and resuscitation, you give pantoprazole, ceftriaxone, and octreotide; by what mechanism does octreotide reduce bleeding in this scenario?
  - Prostaglandin E analogue causes parietal cells to decrease acid production
  - Decreased gastrin production by G cells in pyloric antrum
  - Direct pressure against varices causing tamponade
  - Splanchnic vasoconstriction and decreased portal inflow
6. A 64-year-old male with a history of diabetes, hypertension, and thoracic aortic aneurysm presents to the emergency department for left lower quadrant pain and diarrhea. He is found to have uncomplicated diverticulitis on CT scan; which regimen is most appropriate for this patient?
  - Trimethoprim/sulfamethoxazole
  - Vancomycin PO
  - Metronidazole and ciprofloxacin
  - Amoxicillin/clavulanate
7. A 52-year-old female with a history of alcohol abuse complicated by cirrhosis presents for fever, abdominal pain, jaundice, and confusion. She has a history of anaphylaxis to ceftriaxone; which of the following empiric therapies would you initiate?
  - Ciprofloxacin 400mg BID
  - Cefotaxime 2g q8hrs
  - Ampicillin 2g q4 hrs
  - Telavancin 7.5 mg/kg q 24 hrs
8. A 58-year-old man with a history of well controlled HIV, hypertension and diabetes presents to the ED with severe left upper quadrant pain, nausea, and vomiting. Lipase is elevated at 600 U/L. On CT imaging he has no gallstones and he does not drink alcohol. Which of his medications is the most likely the culprit?
  - Metformin
  - Didanosine
  - Raltegravir
  - Emtricitabine
  - Amlodipine
9. A 60-year-old female presents to the ED for cramping abdominal discomfort and persistent diarrhea for 2 weeks after completing a course of clindamycin for cellulitis. She is well-appearing with normal vital signs. What initial therapy would you start her on?
  - Metronidazole PO
  - Vancomycin IV
  - Vancomycin PO
  - Probiotics
10. A 26-year-old female presents to your ED for 3 days of fever, abdominal pain and bloody diarrhea. She recently returned from a 1-month business trip to Bangkok. Vital signs are HR 112, BP 96/62 T 38.7°C RR 19. In addition to IV hydration and analgesia, which empiric antibiotic therapy would be most appropriate?
  - Levofloxacin
  - Azithromycin
  - Amoxicillin/clavulanate
  - Cefuroxime and metronidazole
11. A 2-year-old male is brought in by his mother for persistent nocturnal perianal itching. Scotch tape test is positive. What is appropriate empiric therapy?
  - Mebendazole
  - Praziquantel
  - Ceftriaxone
  - Metronidazole
12. A 36-year-old female with a history of cholelithiasis presents with fever, jaundice, right upper quadrant abdominal pain. Vitals are normal. In addition to source control, which of the following is an appropriate monotherapy for this process?
  - Piperacillin tazobactam
  - Ceftriaxone
  - Cefotaxime
  - Metronidazole
  - Cefepime
13. A 44-year-old female with chronic epigastric pain, recurrent ulcers, a chronic microcytic anemia and a positive urease breath test as an outpatient presents for worsening of her epigastric abdominal pain. She recently took a Zpack for her sinuses from urgent care. What is the most appropriate antibiotic regimen in this patient?
  - Acetaminophen and Maalox
  - Bismuth, famotidine and metronidazole
  - Clarithromycin, amoxicillin, omeprazole
  - Bismuth subsalicylate, metronidazole, tetracycline, esomeprazole

### Hematology and Oncology Block Questions

1. 5-year-old boy presents to CHP with sudden onset of bright red blood per rectum. T: 37°C, RR: 20, BP: 90/60, HR: 110, Pulse ox: 98%. The patient does not appear to be in any acute distress. Physical exam shows a benign abdomen but is remarkable for gross red blood present in the patient’s underwear. Mother states that the patient was previously being monitored by a hematologist for “low platelet count.” CBC shows platelet count of 29,000. What medication(s) should be started?
  - Transfuse platelets
  - High dose IV steroids
  - IVIG (Intravenous Immunoglobulin)
  - All of the above
2. A 19-year-old male presents to the ED after falling off a ladder at work. He responds to his name but does not know where he is and appears confused. Physical exam reveals a hematoma near the occiput. Quick chart review shows the patient has known hemophilia A. What treatment should be started immediately?
  - 25 IU/kg of Factor VIII concentrate
  - 25 IU/kg of factor IX concentrate
  - 50 IU/kg of Factor VIII concentrate
  - 50 IU/kg of Factor IX concentrate
    If the first line therapy is not available at your hospital, what is the best alternative?
  - Fresh frozen plasma (FFP)
  - Tranexamic Acid
  - Cryoprecipitate
  - Vitamin K
3. A 37-year-old female with history of systemic lupus erythematous presents to the ED with persistent epistaxis for 3 hours. She also notes fatigue and abdominal pain for several days. T:38.1°C, HR: 110, RR: 18, BP: 110/70, Pulse Ox: 99%. Physical exam reveals several purpura on the legs and back and blood oozing from the nasal septum. Labs are remarkable for hemoglobin of 7.1, Platelets 5000, and Creatinine of 2.3. What is the appropriate treatment?
  - Transfuse platelets
  - High dose steroids
  - Plasma exchange
  - All of the above
  - B and C only
4. A 29-year-old female with sickle cell disease presents for chest pain and fever; x-ray shows right lower lobe infiltrate. What is appropriate empiric antibiotic therapy?
  - Piperacillin tazobactam
  - Moxifloxacin
  - Ceftriaxone and azithromycin
  - Cefepime and Vancomycin
5. A 19-year-old male with a history of sickle cell disease presents to the ED for severe pain in both arms and legs. He has recently run out of his hydroxyurea. What is the mechanism by which this drug decreases the frequency of vaso-occlusive crises?
  - Increasing hemoglobin F formation
  - Alkalizing blood
  - Preventing Hemoglobin S formation
  - Decreasing rate of erythrocyte production
6. A 33-year-old man with ALL (acute lymphocytic leukemia) presents with nausea, vomiting, diarrhea, fatigue, shortness of breath and muscle cramps after starting chemotherapy. Which of the following agents would be used to treat his symptoms by increasing uric acid excretion?
  - Rasburicase
  - Allopurinol
  - Furosemide
  - Dexamethasone
  - Febuxostat
7. A 22-year-old man with a history of non-Hodgkin’s lymphoma status post chemotherapy presents for gradual onset dyspnea. He has no lower extremity edema or jugular veinous distention. He is saturating 92% on room air and has diffuse crackles on exams. Chest x-ray shows reticular shadowing pattern with no vascular prominence of effusion. Which agent is most likely responsible?
  - Vincristine
  - Bleomycin
  - Doxorubicin
  - Vinblastine
8. A 64-year-old man with recent hospitalization for STEMI, who was discharged on carvedilol, lisinopril, aspirin, and clopidogrel presents to your emergency department with confusion, fatigue, a petechial rash, and mucosal bleeding. Which of his new medications is the most likely cause?
  - Lisinopril
  - Clopidogrel
  - Aspirin
  - Lisinopril
9. A 64-year-old female with breast cancer presents to your ED with dyspnea. Multiple subsegmental pulmonary embolisms are found on CT scan and heparin therapy is initiated. While boarding in your ED, the patient attempts to get up to go to the bathroom, falls, strikes her head and is found to have a subdural hematoma. What drug would you use to reverse her anticoagulation?
  - Aminocaproic acid
  - Tranexamic acid
  - Vitamin K
  - DDAVP
  - Protamine sulfate
10. A 99-year-old female with atrial fibrillation on coumadin presents to the ED after an all terrain vehicle (ATV) accident. She is somnolent. CT scan shows large subdural hematoma. Her INR (international normalized ratio) is 6.2. You order 4 Factor Prothrombin complex concentrate (Kcentra). Which clotting factors are contained in this drug?
  - II, VII, IX, X
  - VII, VIII, IX, X
  - II, IV, VII, IX
  - II, III, V

### Infectious Disease Block Questions

1. A 22-year-old female with type I diabetes presents to the emergency department with a rash. You find 2 separate 3 cm areas of fluctuance consistent with abscess without significant surrounding erythema. She is concerned that this may interfere with her ability to practice with her college soccer team. What if any antibiotic would you start?
  - Cephalexin
  - Trimethoprim sulfamethoxazole
  - Cefpodoxime
  - None
  - metronidazole
2. A healthy 2-year-old child presents to the ED for 4 days of worsening facial rash. You note a vesicular rash on an erythematous base with golden colored crusting on the vesicles. Child has age-appropriate vital signs and appears non-toxic. What is first line therapy?
  - Clindamycin 10mg/kg po q 6 hrs
  - Debridement
  - Topical Mupirocin q 8 hrs ×5 days
  - Doxycycline 2mg/kg bid
3. A 72-year-old male with diabetes presents to your ED with complaint of severe perineal pain. He has a temperature of 38.3°C and HR of 112, and all other vital signs are normal. You note erythema without sharp margins, pain out of proportion to exam, and an area of edema that extends beyond the margin of the erythema. In addition to surgical consultation and typical broad spectrum sepsis antibiotic coverage, which additional agent should be added for this presentation?
  - Doxycycline
  - Aztreonam
  - Dicloxacillin
  - Clindamycin
4. A 6-year-old child presents to your emergency department for scalp itching and hair loss. Symptoms started several weeks ago and are worsening. Now there is a boggy, draining, crusted hairless area present on the top of the head. He is well appearing afebrile, and nontoxic. Which therapy is indicated for treatment?
  - Oral cephalexin
  - Topical ketoconazole shampoo
  - Oral griseofulvin
  - Incision and drainage
5. A 26-year-old female presents to the emergency department with four days of worsening right lower quadrant tenderness and vaginal discharge. On exam you note purulent discharge. cervical motion tenderness. and right adnexal tenderness. In addition to ceftriaxone in the emergency department, what oral therapy would you prescribe?
  - Doxycycline 5–7 days
  - Clindamycin 10 days
  - amoxicillin-clavulanate 7 days
  - Doxycycline 14 days
6. A 67-year-old female is brought to the emergency department by her daughter with complaints of fever, headache and confusion. On the exam you note T 38.9°C, HR 124, BP 97/58. The patient is oriented only to person with nuchal rigidity. What is appropriate first line empiric therapy?
  - Ceftriaxone
  - Ceftriaxone and vancomycin
  - Ceftriaxone, vancomycin, ampicillin
  - Cefepime, metronidazole, clindamycin
7. A 44-year-old man with tuberculosis in the intensive phase of treatment presents to the emergency department complaining of fatigue and malaise. Which organ system is most likely to be compromised from his treatment regimen?
  - Hepatic
  - Renal
  - Cardiovascular
  - Neurologic
8. A 33-year-old man presents to the emergency department with 2 months of waxing and waning fevers, rigors, nausea and myalgias. Fevers occur every other day. He travels regularly to southeast Asia for business. What is appropriate therapy for this patient?
  - Chloroquine
  - Artemether-lumefantrine
  - Doxycycline
  - Remdesivir
9. A 17-year-old unvaccinated male is brought to the ER with painful strenuous muscle spasms. He has an open wound sustained several days ago in a barn raising accident. In addition to wound management and immune globulin, what antimicrobial agent should be given?
  - Metronidazole
  - Trimethoprim sulfamethoxazole
  - Hydroxychloroquine
  - Acyclovir
10. A 32-year-old female with a history of HIV presents with headache, fever, and new onset seizure today. CD4 count is 87 and brain MRI shows multiple ring-enhancing lesions. What is the therapy of choice for this condition?
  - Doxycycline
  - HAART therapy
  - Sulfadiazine and pyrimethamine
  - Ceftriaxone and vancomycin
11. Bonus: A 24-year-old man recently returning from Nigeria presents to the ED with a complaint of eye irritation. On a slit lamp exam you note punctate keratitis and wriggling microfilariae. What is the treatment of choice?
  - Ivermectin
  - Quinine
  - Ciprofloxacin drops
  - albendazole

### Musculoskeletal Block Questions

1. A 55-year-old male with history of gout, chronic kidney disease, and diabetes presents with severe right foot pain that started this morning. He is afebrile and vital signs otherwise within normal limits. Physical exam shows a warm, exquisitely tender first metatarsophalangeal joint on the right foot. Exam is otherwise unremarkable. Labs from a PCP appointment last week show a baseline creatinine of 2.3, glucose of 135 and a1c of 6.2%. The patient stopped taking allopurinol several months ago because he had not had a gout flare for over a year. What is the most appropriate medication to start at this time?
  - Allopurinol
  - Colchicine
  - Prednisone
  - Naproxen
  - Warm compress
2. A 23-year-old male with no personal medical history presents with 1 week of joint pain. He states that he began having aching pain in his right wrist and ankle early this week, and now his left knee is particularly painful and swollen. He also endorses generalized malaise and some subjective fever at home. He has been sexually active with several partners in the past 2 months. Temp of 38°C on presentation and vitals otherwise within normal limits. Physical exam reveals tender, swollen left knee, and the dorsal aspect of bilateral wrists is tender to palpation. You notice a painless macule on the palm of the left hand. Arthrocentesis performed on knee and synovial fluid analysis shows opaque fluid with WBC 50,000, gram stain show gram negative cocci. What antibiotics should be started?
  - Cefazolin
  - Ceftriaxone and azithromycin
  - Piperacillin-tazobactam
  - Vancomycin
3. A 70-year-old male presents from home with swelling of the right index finger for several days. He has been soaking and applying topical triple antibiotic ointment without any improvement. He states that several months ago he had a sore on his arm that required two different antibiotic prescriptions before finally getting better. Patient is well appearing, afebrile and vitals are within normal limits. Physical exam suggests a paronychia of the right index finger with a small degree of surrounding cellulitis. In addition to drainage, what antibiotic would be most appropriate?
  - Cephalexin
  - Topical bacitracin
  - Trimethoprim-sulfamethoxazole
  - IV Vancomycin
4. A 31-year-old female with a history of rheumatoid arthritis (RA) presents with several weeks of worsening fatigue. She is afebrile, HR 103 bpm and vital signs otherwise within normal limits. CBC shows Hg of 6.3, WBC of 1,400 and Platelets of 78,000. Which drug commonly used in treatment of RA is the mostly likely cause of these lab findings?
  - Hydroxychloroquine
  - Infliximab (Remicade)
  - Methotrexate
  - Prednisone
5. What is the recommended maximum amount of 1% lidocaine with epinephrine that could be used as a local anesthetic when repairing a complicated laceration in a 10-year-old patient weighing 20kg?
  - 8mLs
  - 80mLs
  - 14mLs
  - 140mLs
6. A 27-year-old male presents to the ED with a 4cm laceration to the left forearm which requires repair. The patient states that he has an allergy to lidocaine. Which alternative anesthetic would be the best choice in this patient?
  - Bupivacaine
  - Lidocaine w/epinephrine
  - prilocaine
  - Procaine
  - Mepivacaine
7. A 42-year-old female with systemic lupus erythematosus (SLE), diabetes, and hypertension on lisinopril, hydroxychloroquine, metformin, insulin, and prednisone, presents to the emergency department with right sided groin pain. She is well appearing and afebrile with normal vital signs. Her pain is gradual in onset and worsening, worse with movement, and weightbearing. Hip and pelvis x-rays are normal on your interpretation. MRI is ordered to confirm the diagnosis. Which of her medications do you suspect to be responsible?
  - Hydroxychloroquine
  - Insulin
  - Lisinopril
  - Metformin
  - Prednisone
8. A 42-year-old man presents to the emergency department with pain in the left forearm. He cut himself while working outside 2 weeks ago and superglued the wound at home. He now has worsening pain, erythema and swelling. X-ray imaging shows bony erosion. What is appropriate empiric therapy for this condition?
  - Vancomycin and cephalexin
  - piperacillin -tazobactam and gentamicin
  - Vancomycin and ceftriaxone
  - Levofloxacin and ampicillin
9. A 9-year-old boy presents to your western Pennsylvania emergency department for his second recurrent episode of left knee pain and swelling. He spends a lot of time outside and had multiple tick bites last summer. On exam he is well appearing but has significant pain, swelling, and limitation of range of motion in the right knee with palpable effusion. He has an age-appropriate ECG and no headache or nerve palsy. What is the recommended oral therapy for this patient’s condition?
  - Doxycycline
  - Ceftriaxone
  - Clarithromycin
  - Trimethoprim sulfamethoxazole
10. A 58-year-old man with cirrhosis and low back pain presents to the emergency department with altered mental status. He is placed on the cardiac monitor and found to be in a wide complex tachycardia. He then begins seizing. Which of his medications for back pain is he most likely suffering from toxicity of?
  - Acetaminophen
  - Diclofenac
  - Hydrocodone
  - Cyclobenzaprine
11. A 61-year-old female presents to the emergency department with sudden onset of severe right lower leg pain and inability to ambulate. When the calf is squeezed there is no observed plantar flexion of the ankle. On medical record review, you note she was seen at an urgent care center last week and treated for sinusitis. Based on her current presentation what medication do you think she was most likely given?
  - Amoxicillin-clavulanate
  - Moxifloxacin
  - Doxycycline
  - Fluticasone
  - Azithromycin
12. A 44-year-old man presents to the emergency department with acute non-traumatic low back pain. He has no neurologic deficits, no fever or history of intravenous drug use, and no history of malignancy. Which of the following medications has the strongest evidence basis for treating these symptoms?
  - Acetaminophen
  - NSAID
  - Opioid
  - Benzodiazepine
  - Non benzodiazepine muscle relaxant

### Pulmonary and Critical Care Block Questions

1. You are caring for a 27-year-old asthmatic patient, presenting for severe dyspnea. Despite multiple doses of nebulized albuterol, subcutaneous epinephrine, and Bipap, the patient continues to struggle to breathe and becomes somnolent; you elect to proceed with rapid sequence intubation (RSI). Which of the following agents may provide additional benefits in addition to facilitating RSI?
  - Etomidate
  - Propofol
  - Ketamine
  - Fentanyl
2. A 79-year-old female presents to your emergency department with fever and altered mental status. Her initial vital signs are T 39.4°C, RR 27 HR 145 BP 65/30. You activate the sepsis power plan; however, despite 30 cc/kg of lactated Ringer’s (LR) her BP remains low 72/38, and she continues to decompensate and is obtunded. You believe she needs intubation. Which of the following induction agents is most likely to exacerbate her existing hypotension?
  - Propofol
  - Midazolam
  - Etomidate
  - Ketamine
3. A 66-year-old male presents to your emergency department as a level 1 stroke alert for sudden onset of right-sided weakness 45 minutes ago. On arrival he is completely obtunded and you prepare for intubation. You would like to use succinylcholine to quickly regain a reliable neurologic exam. Which of the following is not a contraindication to use of succinylcholine?
  - History of myotonic dystrophy
  - His acute stroke
  - A history of 20% TBSA (total body surface area) burns 3 weeks ago
  - History of lower extremity paraplegia from spinal cord injury
  - History of malignant hyperthermia
4. A 45-year-old man presents to the emergency department for facial swelling. He reports this has been going on for two days and has happened 6 or 7 times before and no one knows why. His only medical history is hypertension on amlodipine. He also reports he has had intermittent cramping abdominal pain over the past 2 years. His family history is notable for a sudden death in his mother from “her throat swelling up.” If he begins to have worsening swelling and respiratory compromise, what medication is most likely to treat the underlying pathophysiological mechanism?
  - Idarucizumab
  - C1 inhibitor
  - Methylprednisolone
  - Epinephrine
5. A 2-year-old male presents to your emergency department for a cough. On exam the child is uncomfortable in appearance with a barking cough and audible stridor. He has no fever and is non-toxic in appearance. Lung sounds are clear aside from inspiratory stridor. Which of the following choices is the most appropriate regimen?
  - Nebulized epinephrine
  - IM dexamethasone and nebulized epinephrine
  - Nebulized budesonide and albuterol
  - Heliox
  - Nebulized albuterol
6. A 3-year-old female presents to your freestanding emergency department for fever and respiratory distress. You walk in the room and find a lethargic appearing child, in a tripod position, drooling. Unfortunately, the child begins struggling more to breath and becomes somnolent. You are able to successfully intubate, and post intubation x ray shows the ET tube in appropriate position and a thumbprint sign. While making transfer arrangements, what would best medical management be?
  - Vancomycin and piperacillin-tazobactam
  - Dexamethasone, vancomycin and ceftriaxone
  - Clindamycin
  - Dexamethasone and doxycycline
7. A 19-year-old male presents to the emergency department for wheezing and shortness of breath. His vital signs are RR 45 HR 127 T 37.2°C BP 100/60. On exam he has diffuse inspiratory and expiratory wheeze with decreased air movement and O2 saturation of 92% You initiate therapy with continuous albuterol and ipratropium and dexamethasone. You consider adding IV magnesium. Meta-analyses have demonstrated which of the following patient-oriented outcomes?
  - Decreased need for intubation
  - Increased FEV1 (forced expiratory volume)
  - Decreased hospital admission
  - Decreased need for epinephrine
8. A 28-year-old man with no past medical history presents to the ED with chest pain and cough. Vital signs are T 38.7°C HR 93 RR 19 BP 120/84. On exam he has crackles in the right upper lung field but lungs are otherwise clear to auscultation. He is complaining of a cough with purulent sputum. He reports allergies to penicillin and cefuroxime. Your local antibiogram shows a high resistance rate of strep pneumoniae to azithromycin. What is appropriate antibiotic therapy?
  - Ciprofloxacin
  - Doxycycline
  - Vancomycin and levofloxacin
  - Clindamycin and moxifloxacin
9. A 75-year-old female presents to the emergency department for sudden onset of fever, chills, rigors, malaise and cough. Symptom onset was approximately 36 hours ago. Oseltamivir is prescribed for a presumed influenza infection. Which of the following has been a demonstrated benefit of this treatment?
  - Shorter time to defervescence
  - Decreased requirement for hospital admission
  - Decreased need for intubation
  - Shortened duration of illness by approximately 16 hours
10. A 29-year-old female presents to your emergency department for chest pain, leg swelling and dyspnea. Her past medical history is notable for type 1 diabetes, chronic kidney disease stage IV, Factor V Leiden deficiency. She is also 28 weeks pregnant. Bedside ultrasound demonstrates a lower extremity DVT (deep vein thrombosis). Empiric treatment with heparin is initiated. What medication is most likely to be her definitive anticoagulation therapy?
  - Enoxaparin
  - Rivaroxaban
  - Dabigatran
  - Heparin
11. A 53-year-old Chinese immigrant presents to your emergency department for three weeks of productive cough, now with hemoptysis, as well as fever, malaise, night sweats and a 5 kg unintentional weight loss. Chest x-ray shows a cavitary lesion in the right upper lobe. What is appropriate antibiotic therapy for this patient?
  - Levofloxacin
  - Cefuroxime and azithromycin
  - Vancomycin and cefepime
  - Rifampin, Isoniazid, pyrazinamide, and ethambutol (RIPE)
    In the patient above which chronic disease state would likely require a change in initial therapy?
  - Autoimmune hepatitis
  - Type 1 diabetes mellitus
  - Polycystic kidney disease
  - Cerebral palsy
12. A 3-year-old male presents to the emergency department in status epilepticus having already received one dose of lorazepam in the field by EMS. He is accompanied by his grandmother who is concerned that he may have gotten into some of her medicine she has been taking for the past several months for a lung infection. What treatment should you initiate?
  - Pyridoxine
  - Physostigmine
  - Sodium bicarbonate
  - Fosphenytoin
  - Levetiracetam
13. A 9-year-old female presents to the emergency department for 3 weeks of persistent cough. This was preceded by fever with dry cough and rhinorrhea. Parents report that coughing spells tend to come in bursts followed by a forceful inspiration. After making the correct diagnosis, you assess the need to provide prophylaxis for close contacts. Who would require prophylaxis and with what drug?
  - His 4-year-old brother and his father with doxycycline
  - His father with trimethoprim sulfamethoxazole (TMP/SMx)
  - Anyone in the ED waiting room with azithromycin
  - The ED physician who is 37 weeks pregnant with azithromycin
14. A 45-year-old man with a history of asthma, obesity, coronary artery disease, hypertension, diabetes, and obesity presents to the ED with 28 hours of fevers, chills, malaise, cough and myalgias. You clinically suspect influenza and plan to initiate therapy with oseltamivir. Which physiologic parameter is most important to consider when writing the appropriate treatment regimen?
  - Weight
  - Age
  - Oxygen saturation
  - Renal function
  - Hepatic function
15. A 37-year-old man with a history of cystic fibrosis (CF) presents to the emergency department for shortness of breath and worsening of chronic cough. He has a new 2L oxygen requirement and is febrile at 38.2°C. In addition to broad spectrum and anti-pseudomonal antibiotic coverage, which of the following pharmacologic interventions is most likely to be helpful?
  - Sildenafil
  - Dornase alfa
  - High flow non-humidified oxygen
  - Ipratropium bromide
16. A 68-year-old female with a history of DVT, daily tobacco use, and on hormone replacement therapy presents to the emergency department for chest pain. During her stay she becomes tachycardic, hypotensive and diaphoretic. Bedside ultrasound shows right ventricle dilation and septal bowing. You elect to give TPA (tissue plasminogen activator), what is the dosing in this situation?
  - 1 mg/hr over 24 hrs
  - 50 mg over 2 minutes followed by 50 mg over 15 minutes
  - 100mg over 2 hours
  - 15 mg over 1–2 minutes then 50 mg over 30 min, then 35 mg over 1 hour
    What would your answer be if the patient above suffered from cardiac arrest?
  - 1 mg/hr over 24 hrs
  - 50 mg over 2 minutes followed by 50 mg over 15 minutes
  - 100mg over 2 hours
  - 15 mg over 1–2 minutes then 50 mg over 30 min, then 35 mg over 1 hour

### Trauma Block Questions

1. A 24-year-old male presents to the ED after diving into the shallow end of the pool. Initial vital signs are T 36.4°C HR 84 BP 72/50 O2 sat 97%. Patient is unable to move his arms or legs, but is alert and answers questions. FAST exam is negative. In addition to crystalloid infusion, and further evaluation for thoracoabdominal injuries, what medication should be administered at this time?
  - Vasopressin
  - Tranexamic acid
  - Norepinephrine
  - Dopamine
  - PCC
2. An 84-year-old woman presents to the ED as a level 1 trauma activation after a rollover motor vehicle collision (MVC). On arrival she is hypotensive with a FAST exam positive for fluid in the left upper quadrant. The hospital’s only trauma surgeon is already in the operating room with another patient. In addition to balanced transfusion of blood products, what other agents may be indicated in the treatment of this patient’s condition?
  - Tranexamic acid
  - Calcium Gluconate
  - Albumin
  - Andexanet alfa
3. A 33-year-old man presents to the ED following a 17 foot fall off of a rooftop. Evaluation reveals a fracture to the left calcaneus and you decided to perform a posterior tibial nerve block for analgesia. He reports a previous anaphylactic allergic reaction to lidocaine; which anesthetic may you safely use?
  - Bupivacaine
  - Ropivacaine
  - Mepivacaine
  - Tetracaine
4. A 12-year-old girl with type I diabetes presents to the emergency department 3 hours after being bitten on the dorsum of her hand by the family cat. She has a Penicillin allergy. In addition to local wound care, what therapy would you initiate?
  - Ibuprofen
  - Amoxicillin-clavulanate
  - Metronidazole plus doxycycline
  - Clindamycin plus trimethoprim-sulfamethoxazole
5. A 42-year-old artisan was etching glassware when he spilled a large amount of concentrated acid on his hand. He is in severe pain and his EKG showed QT prolongation. Which agent is most important in his treatment?
  - Calcium gluconate
  - Sodium Bicarbonate
  - IV Normal Saline
  - Potassium Chloride
6. A 22-year-old male presents to the emergency department after a motorcycle accident. After primary and secondary survey and CT imaging of the head, chest, abdomen, pelvis and cervical, thoracic and lumbar spines, his only injury is a Gustilo II open tibia fracture. What is appropriate therapy for this injury?
  - Levofloxacin
  - Cefazolin
  - Vancomycin
  - Piperacillin-tazobactam
7. A healthy 47-year-old male presents to your emergency department with 7% TBSA (total body surface area) deep partial thickness burns. Which of the following topical dressings or treatments is relatively contraindicated in this patient due to a possible delay in healing?
  - Bacitracin and xeroform
  - Mepilex
  - Silvadene
  - Mupirocin and xeroform
8. A 62-year-old diabetic male presents to the emergency department today after finding a nail in his shoe when he took it off today. He has minimal sensation in the bottom of his feet due to neuropathy and is unsure when he may have stepped on this nail. Exam reveals a puncture wound on the plantar surface of the foot and a 1.5 cm area of surrounding erythema. What is appropriate therapy to initiate?
  - Piperacillin tazobactam
  - Vancomycin
  - Doxycycline
  - Cefazolin
9. A 101-year-old female presents to the emergency department with headache and confusion after a ground level fall. Her medications include diltiazem and rivaroxaban for atrial fibrillation. Head CT shows left-sided parietal subdural hematoma w/2 cm of midline shift. Which of the following therapies is most appropriate?
  - Vitamin K IV
  - FFP
  - Protamine Sulfate
  - Prothrombin Complex Concentrate
10. A 24-year-old previously unvaccinated man presents to the emergency department with a deep thigh laceration from a pitchfork used daily in the barn for his animals. What is the appropriate tetanus prophylaxis for this patient?
  - Tdap
  - Td
  - Tetanus immune globulin (Tig)
  - Tdap + Tetanus immune globulin

## INSTRUCTOR MATERIALS

### Cardiology Block Answers

1. A 65-year-old man with a history of hypertension, atrial fibrillation, CHF (congestive heart failure), and depression presents to the emergency department with altered mental status following an intentional ingestion. His EKG shows bidirectional ventricular tachycardia; which of the following medications did he ingest?
  - Diltiazem
  - Labetalol
  - Fluoxetine
  - **Digoxin**
    *Although rare, bidirectional ventricular tachycardia is pathognomonic for digoxin toxicity*.
2. A 67-year-old man with a history of tertiary syphilis presents to the emergency department with chest pain radiating to his shoulder and R arm weakness. After correctly diagnosing his aortic dissection and establishing adequate rate control and analgesia, he remains hypertensive. You elect to start sodium nitroprusside. What is the mechanism of action of this drug?
  - L type Calcium channel blockade
  - Direct relaxation of smooth muscle
  - Dopamine-1 agonist
  - *Release of nitric oxide leading to increased intracellular cGMP (cyclic guanosine monophosphate) formation and smooth muscle relaxation*
  - *correctly describes mechanism of sodium nitroprusside*
  - *describes mechanism of nicardipine*
  - *describes mechanism of hydralazine*
  - *describes fenoldopam*
3. After initiating the initiate treatment in the question above, you remember reading about possible toxicity from this drug in medical school. During an unfortunately long wait for the OR, your patient becomes increasingly altered and develops a lactic acidosis. In addition to stopping the medication, what is the appropriate treatment?
  - **Sodium thiosulfate**
  - Calcium Chloride
  - D50
  - Glucagon
    *The question stem describes cyanide toxicity from sodium nitroprusside; treatment would be sodium thiosulfate. Other choices CaCl: treat hyperkalemia or calcium channel blocker toxicity, D50 for hypoglycemia, glucagon for beta blocker overdose*.
4. A 33-year-old female presents to the emergency department with 7 days of chest pain and exertion dyspnea. An EKG shows diffuse ST elevations. Which of the following treatments has been shown to have an increased rate of recurrence of her disease process?
  - Aspirin
  - **Prednisone**
  - Indomethacin
  - Colchicine
    *Isolated use of steroids for treatment of pericarditis are associated with an increased rate of recurrence of pericarditis. Steroids should only be used in patients with contraindications to NSAIDS and colchicine. They should not be started without specialist consultation or discussion with the patient’s primary care provider*.
5. In which of the following circumstances is procainamide contraindicated for the treatment of wide complex tachycardia?
  - Patient with WPW (Wolff-Parkinson-White) in supraventricular tachycardia
  - Patient successfully cardioverted from ventricular tachycardia
  - **Amitriptyline toxicity**
  - Stable ventricular tachycardia
    *Procainamide is a sodium channel blocker and contraindicated in TCA (tricyclic antidepressant) overdose*.
6. A 47-year-old man with a history of asthma and atrial fibrillation presents to the emergency department in respiratory distress with wheezing and poor air movement after a treatment with albuterol and ipratropium-bromide administered by medics. You start a continuous albuterol treatment and note the patient to be tachycardic and irregular at a rate of 150, consistent with atrial fibrillation with rapid ventricular response. In addition to fluid resuscitation, what treatment would you initiate for rate control?
  - Metoprolol
  - **Verapamil**
  - Adenosine
  - Atropine
    *Beta blockers are not the preferred choice for treatment of a-fib in patients with asthma/COPD because the beta-blockade can potentiate worsening bronchoconstriction. Adenosine is not indicated in the treatment of atrial fibrillation. Atropine would likely worsen the patient’s condition*.
7. A 3-day-old infant presents to your emergency department by EMS for poor feeding and lethargy. On examination you find a cyanotic infant, with poor tone, tachypnea and central cyanosis. IV access is obtained via a scalp IV. Stat portable CXR shows an “egg on a string” with narrow mediastinum and increased pulmonary vascular markings. What treatment should you empirically initiate?
  - **Prostaglandin E at 0.1 mcg/kg/min**
  - Prostaglandin E at 5 mcg/kg
  - Prostacyclin at 25 ng/kg/min
  - Prostacyclin at .02 mcg/kg
  - Norepinephrine at .02 mcg/kg/min
    *The question above is describing a neonate with transposition of the great vessels. This is a ductal dependent lesion, and the patient is becoming increasingly symptomatic as the ductus arteriosus is closing. Prostaglandin E1 is crucial for maintaining patency of the ductus arteriosus and should be infused at 0.1 mcg/kg/min. Apnea is a common side effect and you should be prepared to manage and secure the airway in any patient receiving an infusion of prostaglandin*.
8. A 72-year-old female presents to a rural, critical-access hospital complaining of chest pain. She is clutching her chest and diaphoretic. EKG shows ST elevations in lead II, III, aVF. You evaluate her for initiation of fibrinolytic therapy. Which of the following is an absolute contraindication to the use of fibrinolytics?
  - Large ischemic MCA (middle cerebral artery) stroke 4 months ago with residual weakness
  - **Breast cancer with metastasis to brain**
  - Current use of warfarin
  - SBP (systolic blood pressure) on 210 on arrival to ED
    *The other choices would be relative contraindications to tPA (tissue plasminogen activator); known brain metastases would be absolute contraindication*.
9. A 79-year-old male presents to the emergency department with dyspnea and altered mental status. On arrival vital signs are HR 111 BP 77/50 RR 23 T 37.1°C O2 sat 95% on NRB. Examination reveals 3+ LE edema, 5 second capillary refill with cold extremities, and diffuse rales. You evaluate with bedside echo and see LV (left ventricle) and RV (right ventricle) dilation with EF (ejection fraction) estimated 10–15%. Diagnosing acute decompensated heart failure and cardiogenic shock, you elect to begin inotropic support with milrinone in addition to other therapies. What is the mechanism of action of milrinone?
  - **Phosphodiesterase 3 inhibition**
  - Beta1 and Beta2 agonism
  - D1–D5 agonism
  - Alpha1, Beta1, Beta2 agonism
    *Answer choice A accurately describes mechanism of milrinone*
    *Answer choice B describes the mechanism of isoproterenol*
    *Answer Choice D describes dobutamine*
    *Answer choice C describes dopamine*
10. A 4-year-old female is brought into the emergency department for altered mental status and vomiting. The grandmother’s empty digoxin bottles were found with the child. Which of the following tests is most helpful in establishing a prognosis?
  - **Serum potassium**
  - EKG
  - Serum calcium
  - Lactate
    *Potassium has prognostic value for digoxin overdose*.
11. A tall thin 45-year-old man with a history of blindness presents to the emergency department for sudden onset of tearing chest pain radiating to the back. His vital signs are BP 180/130, Pulse 102, RR 23, temp 36.4°C. You note a thoracic aortic dissection on bedside ultrasound at the sternal notch. Which of the following medications should be initiated first?
  - Fenoldopam
  - **Esmolol**
  - Sodium Nitroprusside
  - Clevidipine
    *Beta blockade indicated to decrease shear stress in case of dissection. Beta blockers decrease shear stress by decreasing both heart rate and blood pressure. Sodium nitroprusside can be used as an adjunct therapy but should not be used first line because it can cause a reflex tachycardia. Calcium channel blockers could be considered in patients with severe asthma/COPD to avoid beta-blockade but a nondihydropyridine such as diltiazem would be preferred because the action at the AV (atrioventricular) node helps prevent reflex tachycardia*.
12. A 24-year-old male presents to your emergency department short of breath after just returning from Santiago, Chile. He has rales throughout pulmonary fields and decreased ejection fraction on bedside echo. You suspect Trypanosoma cruzi infection and initiate empiric treatment with?
  - **Benznidazole**
  - Praziquantel
  - Mefloquine
  - Doxycycline
    *Trypanosoma is a common cause of dilated cardiomyopathy worldwide. The treatment of choice for Trypanosoma cruzi infection is benznidazole*.
13. A 19-year-old male presents to the emergency department for chest pain that is worse when lying down at night. You note a small pericardial effusion on bedside echo. Which of the following treatment regimens is associated with fastest resolution of symptoms and lowest rate of recurrence?
  - Prednisone and colchicine
  - Indomethacin and prednisone
  - **Colchicine and aspirin**
  - Aspirin and indomethacin
    *The question stem describes pericarditis. Colchicine and aspirin are associated with the fast resolution. Prednisone can increase rate of recurrence. Choice D is aspirin and NSAID which would have lower efficacy compared to colchicine and aspirin as well as having increased risk of negative side effects such as GI bleed*.
14. An 89-year-old woman presents to the emergency department for heart palpitations and dizziness. Her vital signs are HR 220 BP 100/60 RR 18 T 37.2°C O2 sat 96%. She is alert and oriented. The patient is placed on the cardiac monitor which shows a rhythm concerning for ventricular tachycardia. Treatment is initiated with amiodarone. Which of the following is not an acute adverse effect of amiodarone?
  - Hypotension
  - Torsades de pointes
  - AV nodal blockade
  - **Pulmonary fibrosis**
    *Pulmonary fibrosis is a long-term complication of amiodarone. The other 3 choices are all possible acute effects of amiodarone*

### ENT and Ophthalmology Block Answers

1. A 64-year-old male presents to the ED complaining of 1.5 days of headache, general malaise, and photophobia. On exam you note a vesicular lesion on the tip of the nose. You perform a slit lamp exam and note pseudodendrites. What is the most important component of treatment for this condition?
  - **Oral acyclovir or valacyclovir**
  - Erythromycin ointment
  - Prednisolone acetate drops
  - Cyclopentolate drops
    *Herpes keratitis requires systemic antiviral: IV if sight threatening, usually with topical steroids; if acute retinal necrosis may need systemic steroids as well*
    *Ramsay Hunt syndrome if present requires antivirals as above as well as steroids*.
2. A 3-year-old male presents to the emergency department with 4 days of fever, irritability, poor oral intake and tugging at his ears. On exam both tympanic membranes are erythematous and bulging. Ocular exam is normal. What is the preferred initial treatment for this condition?
  - Amoxicillin 90mg/kg in two divided doses ×14 days
  - **Amoxicillin 45 mg/kg twice per day** × **5–7 days**
  - Amoxicillin/clavulanate 80mg/kg/day dosed by amox in bid divided dosing ×7–10 days
  - Cefpodoxime 10mg/kg × 7 days
    *Stem describing otitis media; amoxicillin is first line therapy, extended 14-day course not necessary. Amoxicillin clavulanate or cefpodoxime reasonable if first line therapy not successful*.
3. If the patient in the above question also had signs of conjunctivitis, what would the preferred treatment be?
  - Azithromycin 10mg/kg per day × 5 days
  - Clindamycin 10 mg/kg TID × 7 days
  - **Amoxicillin/clavulanate 45 mg/kg BID dosed by amoxicillin** ×**7 days**
  - Penicillin 25 mg/kg TID ×10 days
    *Amoxicillin 80–90/kg in divided bid dosing 10 days peds*
    *If amoxicillin has been used in last 30 days, Augmentin, cefdinir, or cefuroxime should be considered*
    *Otitis w/conjunctivitis Augmentin because Haemophilus influenzae most common*.
4. A 15-year-old female presents to the ED complaining of right ear pain. She is a member of the swim team and is self-conscious of her ear wax and uses q-tips 5 times daily. On exam you note tenderness of the tragus, otorrhea, edema and erythema of the auditory canal and a small perforation of the tympanic membrane. What is the preferred treatment for this condition?
  - **Ofloxacin otic drops**
  - Ciprofloxacin hydrocortisone drops
  - Neomycin/polymyxin B/hydrocortisone suspension
  - Ciprofloxacin PO
    *Stem describes otitis externa*
    *First line therapy typically acetic acid-hydrocortisone for mild disease*
    *Stem describes more of a moderate disease; treat with ciprofloxacin-hydrocortisone, ofloxacin, ciprofloxacin-dexamethasone or neomycin-polymyxin B-hydrocortisone*
    *Cipro-hydrocortisone is not recommended if the tympanic membrane is perforated*.
5. If the patient in the above question was a poorly controlled type I diabetic with leukemia on chemotherapy, what treatment would you prescribe?
  - Ofloxacin drops
  - **Ciprofloxacin po**
  - Clindamycin
  - Cefpodoxime
    *For an immunocompromised patient, systemic therapy is recommended, and IV antibiotics are required for malignant otitis externa*.
6. A 17-year-old female with 7 ear piercings, including two high chondral piercings, presents to the ED with severe auricular ear pain, with erythema. What is the treatment of choice?
  - Amoxicillin clavulanate
  - Clindamycin
  - **Ciprofloxacin**
  - Ceftriaxone
    *Stem describes perichondritis, commonly caused by pseudomonas, treated with fluoroquinolone*.
7. A 22-year-old previously healthy, immunized male presents to the ED with right ear pain and swelling, fever and pain in the ear as well as the surrounding area, who has now developed an asymmetric smile on the right side. Examination shows a dull bulging TM (tympanic membrane) as well as an erythematous, boggy, tender mastoid. What is the empirical treatment for this condition?
  - **Vancomycin and ceftriaxone**
  - Ampicillin
  - Vancomycin and gentamicin
  - Piperacillin-tazobactam
    *The stem above describes mastoiditis for which vancomycin and ceftriaxone are the empiric therapy. The most common organisms are strep and staph*.
8. A 72-year-old male with a history of CAD (cerebral artery disease) and TIA (transient ischemic attack) on aspirin and Plavix presents with persistent epistaxis. After failing direct pressure and oxymetazoline, you elect to proceed with packing with a pledget soaked in TXA (tranexamic acid). What is the mechanism of action of this agent?
  - **Inhibits fibrinolysis by displacing plasminogen from fibrin**
  - Alpha 1 receptor agonist causing vasoconstriction
  - Provides an increase in levels of factors II, VII, IX, and X
  - Direct activator of thrombin
    *Choice A accurately describes mechanism of TX*
    *Choice B describes mechanism of phenylephrine*
    *Choice C of Kcentra*
    *Choice D: factor Xa of clotting cascade activates prothrombin to thrombin; there are pharmaceuticals that inhibit, but not enhance this process*.
9. A 48-year-old female presents to the ED for 5 days of constant facial pain, nasal congestion and pressure, worse with bending over, and subjective fever. On evaluation patient is nontoxic, temp 37.9°C, with tenderness to palpation over maxillary sinuses. Which is the most reasonable treatment for this condition?
  - Amoxicillin-clavulanate
  - Levofloxacin
  - Azithromycin
  - **Fluticasone nasal spray and saline irrigation**
    *Stem describes acute rhinosinusitis*
    *Most cases are viral and can be treated as such*
    *If less than 10 days of symptoms without alarm symptoms, antibiotics are not indicated and medications such as oxymetazoline and fluticasone nasal spray should aid in symptom relief*
    *If second sickening, toxic appearance, or prolonged symptoms, treat with antimicrobials, such as Augmentin*.
10. A 22-year-old female presents to your ER with throat pain. Vital signs are HR 109, T 38.6°C, BP 110/72, RR 20. On examination she has tonsillar exudate w/asymmetrical swelling R>L and tender cervical LAD (lymphadenopathy), and two finger trismus. A CT scan shows cellulitis with no discrete fluid collection. Her allergy list includes penicillin (hives), and hydrocodone (vomiting). Which curative therapy would you initiate?
  - Ampicillin- sulbactam
  - **Clindamycin**
  - Doxycycline
  - Moxifloxacin
    *Stem describes peritonsillar cellulitis*
    *First line empiric therapy amoxicillin-clavulanate (allergy) or clindamycin*
    *If parenteral needed, ampicillin-sulbactam or clindamycin; consider community prevalence of MRSA (methicillin-resistant Staphylococcus aureus) for need of additional coverage*.
11. A 21-year-old male presents to the emergency department 4 days after wisdom tooth extraction with severe pain. Pain had initially subsided before acute worsening. Vital signs are within normal limits including temperature. On examination you see exposed bone with no visible clot in the extraction site. Which of the following should be included in the treatment regimen?
  - Biting down on black tea bags
  - Trimethoprim-sulfamethoxazole
  - **Iodoform gauze soaked in clove oil**
  - Ampicillin-sulbactam
    *The stem describes alveolar osteitis “dry socket.” Clove oil can help symptoms and antibiotics not required. Tea bags can be useful for dental bleeding, but are not indicated here*.
12. A 47-year-old male, who does not wear contact lenses, presents to the emergency department for the second time in 2 weeks. At his first visit he was found to have an R facial palsy that did not spare the forehead. Today he presents for severe eye pain and decreased visual acuity. Exam reveals a corneal defect w/fluorescein uptake, consensual photophobia, and trace hypopyon. What treatment regimen should be initiated?
  - Erythromycin ointment three times daily
  - **Moxifloxacin drops q1 hour**
  - Ciprofloxacin drops twice daily
  - Trifluridine drops twice daily
  - Cyclopentolate twice daily
    *Stem describing corneal ulcer. Needs q1hr quinolone drops and ophthalmology consultation*.
13. A 23-year-old female presents to the ED with right eye pain and redness following a direct blow to the orbit. On examination she has eye pain not relieved by tetracaine. She also has direct and consensual photophobia. Visual acuity 20/100 OD compared to 20/30 OS. Cyclopentolate drops are started as part of her treatment regimen. What is the mechanism of this drug?
  - Sympathomimetic causing cycloplegia and mydriasis
  - Topical anesthetic blocking sodium channels
  - Steroid decreasing inflammation
  - **Anticholinergic causing mydriasis and cycloplegia**
    *Stem describes traumatic iritis; choice D appropriately describes mechanism*.
14. A 48-year-old female with a history of sickle cell disease presents to the emergency department for sudden onset of right-sided eye pain, severe headache, and blurry vision. On evaluation she is noted to have ciliary flush and pupil is fixed and midrange intraocular pressure is 42. Which of the following is contraindicated in the immediate management of this patient?
  - Topical timolol
  - **Systemic acetazolamide**
  - Topical brimonidine
  - Topical pilocarpine
    *The stem describes acute angle closure glaucoma. All medications could be part of general treatment for this disease. However, systemic acetazolamide is contraindicated in patients with sickle cell disease because acetazolamide increases the risk of further sickling of red blood cells*.
15. A 54-year-old female with history of poorly controlled diabetes presents to the emergency department with 2 days of sudden onset and rapidly progressive severe nasal, facial, periorbital pain and headache. She has noticed double vision and discoloration of her nose as well. On a physical exam you also notice black discoloration of the palate as well as inability to fully abduct the right eye. In addition to surgical consultation what treatment should you initiate?
  - Fluconazole
  - **Amphotericin**
  - Vancomycin and piperacillin and tazobactam
  - Clindamycin, ceftriaxone
    *The stem is describing mucormycosis, and amphotericin is the empiric therapy*.

### Gastroenterology Block Answers

1. A 6-year-old female presents to the emergency department for parental concern over white spots in her mouth. She is well appearing and in no apparent distress. On exam you note white plaques in the oropharynx that scrape off with a tongue depressor. What treatment is most appropriate for this condition?
  - Fluconazole po
  - Liposomal amphotericin swish and spit
  - Chlorhexidine rinses
  - **Clotrimazole troche**
    *Oral candidiasis, clotrimazole troche or nystatin swish and swallow; UpToDate recommends clotrimazole over nystatin, but generally both recommended*.
2. A 28-year-old undomiciled male with HIV presents to the emergency department. He has been unable to fill anti-retroviral prescriptions and is complaining of chest pain, dysphagia, hematemesis and fever. His last CD4 count was 12. On examination patient appears very uncomfortable requiring multiple doses of analgesia. EKG and CXR are within normal limits and on examination no thrush or white plaques are seen. What treatment is most likely to be effective?
  - **Ganciclovir**
  - Fluconazole
  - Pantoprazole
  - Aspirin
  - Ceftriaxone
    *CMV (cytomegalovirus) esophagitis is treated with valganciclovir or ganciclovir*.
3. A 74-year-old man with severe osteoarthritis of the knees presents with epigastric pain and melena. He appears pale, with a heart rate of 104 and hemoglobin of 5.8. Which of the following agents taken daily is most likely responsible for this presentation?
  - Acetaminophen
  - **Diclofenac**
  - Oxycodone
  - Lavender oil
4. What is the appropriate initial antisecretory therapy for the patient above?
  - Famotidine
  - **Pantoprazole**
  - Sucralfate
  - Magnesium hydroxide + aluminum hydroxide
  - Octreotide
    *Diclofenac is an NSAID which increases the risk of GI bleed. Protein pump inhibitors are used in GI bleed because they will increase the pH of the stomach allowing the clotting factors to form a more stable clot. Clotting factors are proteins and become denatured at lower pH’s. So acute administration allows for a better clot to form and has been shown to reduce blood product administration*.
5. A 37-year-old male with a long-standing history of alcohol abuse presents for massive hematemesis. In addition to airway management and resuscitation, you give pantoprazole, ceftriaxone, and octreotide; by what mechanism does octreotide reduce bleeding in this scenario?
  - Prostaglandin E analogue causes parietal cells to decrease acid production
  - Decreased gastrin production by G cells in pyloric antrum
  - Direct pressure against varices causing tamponade
  - **Splanchnic vasoconstriction and decreased portal inflow**
    *Choice D describes mechanism of action of octreotide appropriately*
    *Choice a describes the mechanism of misoprostol*
    *Choice b describes the mechanism of H2 blockers such as famotidine*
    *Choice c describes the mechanism of a balloon tamponade device*
6. A 64-year-old male with a history of diabetes, hypertension, and thoracic aortic aneurysm presents to the emergency department for left lower quadrant pain and diarrhea. He is found to have uncomplicated diverticulitis on CT scan; which regimen is most appropriate for this patient?
  - Trimethoprim/sulfamethoxazole
  - Vancomycin PO
  - Metronidazole and ciprofloxacin
  - **Amoxicillin/clavulanate**
    *The question stem is describing diverticulitis. Appropriate treatment would be Augmentin as above or cephalosporin and metronidazole. However, given the patient’s history of aortic aneurysm, you should avoid a fluoroquinolone if possible*.
7. A 52-year-old female with a history of alcohol abuse complicated by cirrhosis presents for fever, abdominal pain, jaundice, and confusion. She has a history of anaphylaxis to ceftriaxone; which of the following empiric therapies would you initiate?
  - **Ciprofloxacin 400mg BID**
  - Cefotaxime 2g q8hrs
  - Ampicillin 2g q4 hrs
  - Telavancin 7.5 mg/kg q 24 hrs
    *The stem describes spontaneous bacterial peritonitis. Ciprofloxacin is the alternative regimen for cephalosporin allergy*.
8. A 58-year-old man with a history of well controlled HIV, hypertension and diabetes presents to the ED with severe left upper quadrant pain, nausea, and vomiting. Lipase is elevated at 600 U/L. On CT imaging he has no gallstones and he does not drink alcohol. Which of his medications is the most likely the culprit?
  - Metformin
  - **Didanosine**
  - Raltegravir
  - Emtricitabine
  - Amlodipine
    *The question stem describes a patient with a clinical presentation concerning for pancreatitis*
    *Didanosine is classically associated with drug-induced pancreatitis with an overall frequency of pancreatitis around 6–7% with monotherapy. Elderly patients are at an increased risk of pancreatitis related to didanosine*
    *Metformin has case reports associated with pancreatitis classified as possible cause of drug-induced pancreatitis, but less likely. The list of drugs associated with pancreatitis is very large and needs careful evaluation for other more common causes of pancreatitis*.
9. A 60-year-old female presents to the ED for cramping abdominal discomfort and persistent diarrhea for 2 weeks after completing a course of clindamycin for cellulitis. She is well-appearing with normal vital signs. What initial therapy would you start her on?
  - Metronidazole PO
  - Vancomycin IV
  - **Vancomycin PO**
  - Probiotics
    *The stem is describing Clostridium difficile infection. First line therapy is now po vancomycin, was formerly po metronidazole for mild disease. Oral fidaxomicin other option*.
10. A 26-year-old female presents to your ED for 3 days of fever, abdominal pain and bloody diarrhea. She recently returned from a 1-month business trip to Bangkok. Vital signs are HR 112, BP 96/62 T 38.7°C RR 19. In addition to IV hydration and analgesia, which empiric antibiotic therapy would be most appropriate?
  - Levofloxacin
  - **Azithromycin**
  - Amoxicillin/clavulanate
  - Cefuroxime and metronidazole
    *The stem is describing concern for infectious diarrhea*
    *Indication for antibiotic therapy severe disease: fever, volume depletion requiring hospitalization. Blood or mucoid stools. Age >70 or extensive comorbid risk factors. Recommendation of macrolide or quinolone for infectious diarrhea, and there are high rates of quinolone resistance in SE Asia. Common etiologies to consider include salmonella, campylobacter, shigella, E coli, protozoa and viral causes*.
11. A 2-year-old male is brought in by his mother for persistent nocturnal perianal itching. Scotch tape test is positive. What is appropriate empiric therapy?
  - **Mebendazole**
  - Praziquantel
  - Ceftriaxone
  - Metronidazole
    *The stem describes scotch tape test for pruritus ani. Positive test showing evidence of pinworm, enterobius, so treatment with mebendazole. Albendazole or pyrantel pamoate can also be considered*.
12. A 36-year-old female with a history of cholelithiasis presents with fever, jaundice, right upper quadrant abdominal pain. Vitals are normal. In addition to source control, which of the following is an appropriate monotherapy for this process?
  - **Piperacillin tazobactam**
  - Ceftriaxone
  - Cefotaxime
  - Metronidazole
  - Cefepime
    *The stem is describing ascending cholangitis (Charcot’s triad). Cephalosporins can be used in conjunction with metronidazole but are not appropriate monotherapy because they do not offer adequate anaerobic coverage*.
13. A 44-year-old female with chronic epigastric pain, recurrent ulcers, a chronic microcytic anemia and a positive urease breath test as an outpatient presents for worsening of her epigastric abdominal pain. She recently took a Zpack for her sinuses from urgent care. What is the most appropriate antibiotic regimen in this patient?
  - Acetaminophen and Maalox
  - Bismuth, famotidine and metronidazole
  - Clarithromycin, amoxicillin, omeprazole
  - **Bismuth subsalicylate, metronidazole, tetracycline, esomeprazole**
    *The stem is describing peptic ulcer disease secondary to H. Pylori*
    *Bismuth quadruple therapy recommended given recent macrolide exposure*
    *Answer choice C is a reasonable option in someone without recent macrolide use*.

### Hematology and Oncology Block Answers

1. 5-year-old boy presents to CHP with sudden onset of bright red blood per rectum. T: 37°C, RR: 20, BP: 90/60, HR: 110, Pulse ox: 98%. The patient does not appear to be in any acute distress. Physical exam shows a benign abdomen but is remarkable for gross red blood present in the patient’s underwear. Mother states that the patient was previously being monitored by a hematologist for “low platelet count.” CBC shows platelet count of 29,000. What medication(s) should be started?
  - Transfuse platelets
  - High dose IV steroids
  - IVIG (Intravenous Immunoglobulin)
  - **d. All of the above**
    *GI bleed is considered a severe form of bleeding in ITP (idiopathic thrombocytopenic purpura) and should be treated aggressively, especially when platelet count is less than 30,000. Platelet transfusion serves to immediately increase platelet count. Steroids help support platelet count by immunosuppression which results in decreased antibody production and platelet destruction via the reticulo-endothelial system. IVIG also helps prevent destruction of platelets by the spleen*.
2. A 19-year-old male presents to the ED after falling off a ladder at work. He responds to his name but does not know where he is and appears confused. Physical exam reveals a hematoma near the occiput. Quick chart review shows the patient has known hemophilia A. What treatment should be started immediately?
  - 25 IU/kg of Factor VIII concentrate
  - 25 IU/kg of factor IX concentrate
  - **50 IU/kg of Factor VIII concentrate**
  - 50 IU/kg of Factor IX concentrate
    *There is a concern for intracranial bleed in this setting. Major bleeding in hemophilia (intracranial hemorrhage, preoperative bleeding, compartment syndrome) should be treated with an initial goal for 100% correction of factor activity. Patients with hemophilia are deficient in factor VIII. 1 IU/kg of factor VIII increases activity by approximately 2%, so 50 IU/kg of ideal body weight should be given for life-threatening bleeds*
    If the first line therapy is not available at your hospital, what is the best alternative?
  - Fresh frozen plasma (FFP)
  - Tranexamic Acid
  - **c. Cryoprecipitate**
  - Vitamin K
    *Cryoprecipitate is a second line therapy for heme A if factor XIII is not available. FFP can be used in place of factor IX if not available for hemophilia B*.
3. A 37-year-old female with history of systemic lupus erythematous presents to the ED with persistent epistaxis for 3 hours. She also notes fatigue and abdominal pain for several days. T:38.1°C, HR: 110, RR: 18, BP: 110/70, Pulse Ox: 99%. Physical exam reveals several purpura on the legs and back and blood oozing from the nasal septum. Labs are remarkable for hemoglobin of 7.1, Platelets 5000, and Creatinine of 2.3. What is the appropriate treatment?
  - Transfuse platelets
  - High dose steroids
  - Plasma exchange
  - All of the above
  - **B and C only**
    *The patient is demonstrating signs and symptoms of TTP (thrombotic thrombocytopenic purpura). TTP is oftentimes idiopathic, but it is also associated with autoimmune diseases like lupus. The classic pentad is neurological changes, thrombocytopenia, fever, renal impairment, and hemolytic anemia, but this is only present about 40% of the time. Treatment is plasma exchange with FFP, and steroids are thought to decrease production of ADAMTS13 inhibitors. Platelet transfusion is contraindicated in TTP except in some instances of intracranial hemorrhage*.
4. A 29-year-old female with sickle cell disease presents for chest pain and fever; x-ray shows right lower lobe infiltrate. What is appropriate empiric antibiotic therapy?
  - Piperacillin tazobactam
  - Moxifloxacin
  - **Ceftriaxone and azithromycin**
  - Cefepime and Vancomycin
    *First line treatment for acute chest similar to community-acquired PNA; make sure to cover for atypicals*.
5. A 19-year-old male with a history of sickle cell disease presents to the ED for severe pain in both arms and legs. He has recently run out of his hydroxyurea. What is the mechanism by which this drug decreases the frequency of vaso-occlusive crises?
  - **Increasing hemoglobin F formation**
  - Alkalizing blood
  - Preventing Hemoglobin S formation
  - Decreasing rate of erythrocyte production
    *Hydroxyurea increases fetal hemoglobin (HbF) synthesis and the inhibitory effect of HbF on polymerization of sickle cell hemoglobin*.
6. A 33-year-old man with ALL (acute lymphocytic leukemia) presents with nausea, vomiting, diarrhea, fatigue, shortness of breath and muscle cramps after starting chemotherapy. Which of the following agents would be used to treat his symptoms by increasing uric acid excretion?
  - **Rasburicase**
  - Allopurinol
  - Furosemide
  - Dexamethasone
  - Febuxostat
    *Rasburicase is a recombinant urate-oxidase enzyme, which converts uric acid to allantoin. Allantoin is an inactive and soluble metabolite of uric acid which allows for increased excretion. However, rasburicase does not inhibit the formation of uric acid*.
7. A 22-year-old man with a history of non-Hodgkin’s lymphoma status post chemotherapy presents for gradual onset dyspnea. He has no lower extremity edema or jugular veinous distention. He is saturating 92% on room air and has diffuse crackles on exams. Chest x-ray shows reticular shadowing pattern with no vascular prominence of effusion. Which agent is most likely responsible?
  - Vincristine
  - **Bleomycin**
  - Doxorubicin
  - Vinblastine
    *Oncology side effects. Bleomycin causing pulmonary fibrosis*
    *Vincristine + vinblastine: microtubule inhibitor, classically neuropathy (peripheral)*
    *Doxorubicin: cardiotoxicity*.
8. A 64-year-old man with recent hospitalization for STEMI, who was discharged on carvedilol, lisinopril, aspirin, and clopidogrel presents to your emergency department with confusion, fatigue, a petechial rash, and mucosal bleeding. Which of his new medications is the most likely cause?
  - Lisinopril
  - **Clopidogrel**
  - Aspirin
  - Lisinopril
    *Clopidogrel can cause ITP*
9. A 64-year-old female with breast cancer presents to your ED with dyspnea. Multiple subsegmental pulmonary embolisms are found on CT scan and heparin therapy is initiated. While boarding in your ED, the patient attempts to get up to go to the bathroom, falls, strikes her head and is found to have a subdural hematoma. What drug would you use to reverse her anticoagulation?
  - Aminocaproic acid
  - Tranexamic acid
  - Vitamin K
  - DDAVP
  - **Protamine sulfate**
    *Protamine sulfate reversal agent for heparin*
    *Aminocaproic acid is reversal agent for thrombolytics*
    *Vitamin K reversal for coumadin*
    *DDAVP for von Willebrand or uremic platelet dysfunction*.
10. A 99-year-old female with atrial fibrillation on coumadin presents to the ED after an all terrain vehicle (ATV) accident. She is somnolent. CT scan shows large subdural hematoma. Her INR (international normalized ratio) is 6.2. You order 4 Factor Prothrombin complex concentrate (Kcentra). Which clotting factors are contained in this drug?
  - **II, VII, IX, X**
  - VII, VIII, IX, X
  - II, IV, VII, IX
  - II, III, V
    *These are the four clotting factors found in the drug in question*

### Infectious Disease Block Answers

1. A 22-year-old female with type I diabetes presents to the emergency department with a rash. You find 2 separate 3 cm areas of fluctuance consistent with abscess without significant surrounding erythema. She is concerned that this may interfere with her ability to practice with her college soccer team. What if any antibiotic would you start?
  - Cephalexin
  - **Trimethoprim sulfamethoxazole**
  - Cefpodoxime
  - None
  - metronidazole
    *Given multiple abscesses, diabetes, and likely close skin-to-skin contact/locker room setting. Most abscesses are caused by staph and increasingly MRSA predominant. Let local antibiograms guide you; multiple studies showing efficacy of Bactrim and clindamycin*
    *Recommend antibiotics for abscess if:*
    *>2cm*
    *Multiple lesions*
    *Extensive cellulitis*
    *Comorbidities or immunosuppression*
    *Systemic signs of toxicity*
    *Failed incision and drainage*
    *Hardware*
    *Risk of endocarditis*
    *Or High MRSA risk (they cite athletes and military in UpToDate article)*
2. A healthy 2-year-old child presents to the ED for 4 days of worsening facial rash. You note a vesicular rash on an erythematous base with golden colored crusting on the vesicles. Child has age-appropriate vital signs and appears non-toxic. What is first line therapy?
  - **Clindamycin 10mg/kg po q 6 hrs**
  - Debridement
  - Topical Mupirocin q 8 hrs ×5 days
  - Doxycycline 2mg/kg bid
    *The stem is describing impetigo which can be treated topically. Oral antibiotics are also an option and are indicated if there are numerous lesions, population outbreaks of disease, and ecthyma (deeper and ulcerated)*
3. A 72-year-old male with diabetes presents to your ED with complaint of severe perineal pain. He has a temperature of 38.3°C and HR of 112, and all other vital signs are normal. You note erythema without sharp margins, pain out of proportion to exam, and an area of edema that extends beyond the margin of the erythema. In addition to surgical consultation and typical broad spectrum sepsis antibiotic coverage, which additional agent should be added for this presentation?
  - Doxycycline
  - Aztreonam
  - Dicloxacillin
  - **Clindamycin**
    *Question describes necrotizing fasciitis. Additional clinical signs include crepitus or bullae, or ecchymosis. Primary treatment is surgical, but broad-spectrum antibiotics are also indicated. Clindamycin is thought to help decrease bacterial toxin production via its inhibition of bacterial ribosomes*.
4. A 6-year-old child presents to your emergency department for scalp itching and hair loss. Symptoms started several weeks ago and are worsening. Now there is a boggy, draining, crusted hairless area present on the top of the head. He is well appearing afebrile, and nontoxic. Which therapy is indicated for treatment?
  - Oral cephalexin
  - Topical ketoconazole shampoo
  - **Oral griseofulvin**
  - Incision and drainage
    *Stem describes tinea capitis dermatophyte infection, progressing to kerion. Treatment is systemic antifungals with griseofulvin for 6–12 weeks or terbinafine for 4–6 weeks. Azoles are also effective but not as frequently used and less data behind them. Generally topical treatment indicated as well such as ketoconazole or selenium sulfide shampoo. Kerion does require systemic therapy*.
5. A 26-year-old female presents to the emergency department with four days of worsening right lower quadrant tenderness and vaginal discharge. On exam you note purulent discharge. cervical motion tenderness. and right adnexal tenderness. In addition to ceftriaxone in the emergency department, what oral therapy would you prescribe?
  - Doxycycline 5–7 days
  - Clindamycin 10 days
  - amoxicillin-clavulanate 7 days
  - **Doxycycline 14 days**
    *Question stem refers to PID (pelvic inflammatory disease). Treatment is ceftriaxone and 14 days doxycycline if the patient is stable for discharge. If trichomoniasis or BV is suspected or if patient has had recent instrumentation, metronidazole should also be given (per UpToDate). CDC guidelines take a broader approach with metronidazole for everyone especially if giving 3rd generation cephalosporin. For patients requiring hospitalization and IV antibiotics, use cefoxitin, doxycycline or clindamycin, and gentamicin*.
6. A 67-year-old female is brought to the emergency department by her daughter with complaints of fever, headache and confusion. On the exam you note T 38.9°C, HR 124, BP 97/58. The patient is oriented only to person with nuchal rigidity. What is appropriate first line empiric therapy?
  - Ceftriaxone
  - Ceftriaxone and vancomycin
  - **Ceftriaxone, vancomycin, ampicillin**
  - Cefepime, metronidazole, clindamycin
    *Patient presented is highly concerning for meningitis: ceftriaxone covers typical bacteria, vancomycin for resistant pneumococcus, ampicillin for age over 50 needing listeria coverage*.
7. A 44-year-old man with tuberculosis in the intensive phase of treatment presents to the emergency department complaining of fatigue and malaise. Which organ system is most likely to be compromised from his treatment regimen?
  - **Hepatic**
  - Renal
  - Cardiovascular
  - Neurologic
    *Standard of care for TB treatment is “RIPE” therapy. Rifampin, Isoniazid, pyrazinamide, and ethambutol. Aside from ethambutol, all of these drugs are associated with hepatotoxicity and baseline liver function tests should be taken before initiation of therapy*.
8. A 33-year-old man presents to the emergency department with 2 months of waxing and waning fevers, rigors, nausea and myalgias. Fevers occur every other day. He travels regularly to southeast Asia for business. What is appropriate therapy for this patient?
  - Chloroquine
  - **Artemether-lumefantrine**
  - Doxycycline
  - Remdesivir
    *The presentation above is concerning for malaria. Assume P. falciparum infection. In large areas of the world, including Africa and SE Asia, malaria is chloroquine resistant, and artemisium-based combination therapy is standard*.
9. A 17-year-old unvaccinated male is brought to the ER with painful strenuous muscle spasms. He has an open wound sustained several days ago in a barn raising accident. In addition to wound management and immune globulin, what antimicrobial agent should be given?
  - **Metronidazole**
  - Trimethoprim sulfamethoxazole
  - Hydroxychloroquine
  - Acyclovir
    *Stem describing tetanus. Metronidazole or Penicillin G standard of care. One could also use cephalosporin, especially if concerned for polymicrobial infection*
    *Doxycycline, macrolides, clindamycin, vancomycin, chloramphenicol may work, but lack in vitro data*.
10. A 32-year-old female with a history of HIV presents with headache, fever, and new onset seizure today. CD4 count is 87 and brain MRI shows multiple ring-enhancing lesions. What is the therapy of choice for this condition?
  - Doxycycline
  - HAART therapy
  - **Sulfadiazine and pyrimethamine**
  - Ceftriaxone and vancomycin
    *This presentation is highly concerning for toxoplasmosis encephalitis which is treated with sulfadiazine and pyrimethamine. Leucovorin should be considered as well to prevent hematologic toxicity. Clindamycin and pyrimethamine is an option for sulfa allergy*.
11. Bonus: A 24-year-old man recently returning from Nigeria presents to the ED with a complaint of eye irritation. On a slit lamp exam you note punctate keratitis and wriggling microfilariae. What is the treatment of choice?
  - **Ivermectin**
  - Quinine
  - Ciprofloxacin drops
  - albendazole
    *The question stem is describing Onchocerciasis. To optimize visualization, have the patient lean forward for 2 minutes prior to the slit lamp exam. Punctate keratitis is from dead microfilariae. Ivermectin is standard therapy. However, doxycycline is an emerging treatment, although it does not kill microfilariae so ivermectin should also be given. In endemic areas, loa loa co-infection is common and albendazole should also be considered*
    *Ivermectin binds to glutamate gated chloride channels in invertebrate muscle and nerve cells that causes hyperpolarization and death*.

### Musculoskeletal Block Answers

1. A 55-year-old male with history of gout, chronic kidney disease, and diabetes presents with severe right foot pain that started this morning. He is afebrile and vital signs otherwise within normal limits. Physical exam shows a warm, exquisitely tender first metatarsophalangeal joint on the right foot. Exam is otherwise unremarkable. Labs from a PCP appointment last week show a baseline creatinine of 2.3, glucose of 135 and a1c of 6.2%. The patient stopped taking allopurinol several months ago because he had not had a gout flare for over a year. What is the most appropriate medication to start at this time?
  - Allopurinol
  - Colchicine
  - **Prednisone**
  - Naproxen
  - Warm compress
    *Colchicine, NSAIDs, and corticosteroids have all been shown to be efficacious in treatment of acute gouty flares. However, this patient has chronic kidney disease, and therefore NSAIDs should be avoided. Additionally, colchicine has a higher risk of toxicity in renally insufficient patients. Although diabetic, the patient appears well controlled and a short course of steroids is the best option given the patient’s comorbidities. Initiation of allopurinol or other urate lowering drugs should be avoided in acute flares because they can exacerbate symptoms. However, if the patient is on routine urate lowering drug therapy, they should continue prescribed dosing without interruption. Warm compress may also exacerbate symptoms*.
2. A 23-year-old male with no personal medical history presents with 1 week of joint pain. He states that he began having aching pain in his right wrist and ankle early this week, and now his left knee is particularly painful and swollen. He also endorses generalized malaise and some subjective fever at home. He has been sexually active with several partners in the past 2 months. Temp of 38°C on presentation and vitals otherwise within normal limits. Physical exam reveals tender, swollen left knee, and the dorsal aspect of bilateral wrists is tender to palpation. You notice a painless macule on the palm of the left hand. Arthrocentesis performed on knee and synovial fluid analysis shows opaque fluid with WBC 50,000, gram stain show gram negative cocci. What antibiotics should be started?
  - Cefazolin
  - **Ceftriaxone and azithromycin**
  - Piperacillin-tazobactam
  - Vancomycin
    *This presentation is highly concerning for disseminated gonococcal infection (DGI). Classic presentation involves dermatitis, tenosynovitis and migratory polyarthritis. Thus, antibiotic coverage should involve a third-generation cephalosporin as well as co-treatment for chlamydia. Zosyn does not cover chlamydia. Although gram stain/culture is often negative in DGI, gram stain does reveal gram-negative cocci in this instance and cefazolin and vancomycin do not provide adequate coverage*.
3. A 70-year-old male presents from home with swelling of the right index finger for several days. He has been soaking and applying topical triple antibiotic ointment without any improvement. He states that several months ago he had a sore on his arm that required two different antibiotic prescriptions before finally getting better. Patient is well appearing, afebrile and vitals are within normal limits. Physical exam suggests a paronychia of the right index finger with a small degree of surrounding cellulitis. In addition to drainage, what antibiotic would be most appropriate?
  - Cephalexin
  - Topical bacitracin
  - **Trimethoprim-sulfamethoxazole**
  - IV Vancomycin
    *While recommendations for treatment of paronychia vary some, oral antibiotic therapy is warranted in this circumstance because the patient has continued symptoms after conservative treatment at home and evidence of surrounding cellulitis. Most common pathogens involved in paronychia include staph and strep. Cephalexin offers sufficient coverage most of the time. However, this patient mentioned a previous resistant infection that was likely MRSA. As the patient is otherwise well appearing and afebrile, patient should be managed in the outpatient setting with PO antibiotics making IV vancomycin less appropriate*.
4. A 31-year-old female with a history of rheumatoid arthritis (RA) presents with several weeks of worsening fatigue. She is afebrile, HR 103 bpm and vital signs otherwise within normal limits. CBC shows Hg of 6.3, WBC of 1,400 and Platelets of 78,000. Which drug commonly used in treatment of RA is the mostly likely cause of these lab findings?
  - Hydroxychloroquine
  - Infliximab (Remicade)
  - **Methotrexate**
  - Prednisone
    *Although a rare side effect and most associated with large doses, methotrexate can cause pancytopenia and bone marrow suppression, and this is a boxed warning for methotrexate. Infliximab can be associated with anemia, but this is more common in pediatric populations, and it does not typically cause dysfunction across all three cell lines. Hydroxychloroquine can rarely cause bone marrow suppression but this is much less common than with methotrexate. Although prednisone can cause immunosuppression, it does not typically cause bone marrow suppression*.
5. What is the recommended maximum amount of 1% lidocaine with epinephrine that could be used as a local anesthetic when repairing a complicated laceration in a 10-year-old patient weighing 20kg?
  - 8mLs
  - 80mLs
  - **14mLs**
  - 140mLs
    *The maximum dosing of lidocaine is 4mg/kg for lidocaine without epinephrine and 7mg/kg for lidocaine with epinephrine. It is important to remember that a 1% lidocaine solution has 10mg of lidocaine per 1mL of solution. Thus*

$$\frac{\frac{7mg}{kg}\times 20kg}{10mg/mL}=14mLs$$

- 6. A 27-year-old male presents to the ED with a 4cm laceration to the left forearm which requires repair. The patient states that he has an allergy to lidocaine. Which alternative anesthetic would be the best choice in this patient?
  - Bupivacaine
  - Lidocaine w/epinephrine
  - prilocaine
  - **Procaine**
  - Mepivacaine
    *Lidocaine is among the amide classification of anesthetics. Bupivacaine, prilocaine, and mepivacaine are also all amides which means the patient may likely be allergic to these as well. A helpful memory aid can be that amides all have 2 “I”s in the name. Procaine (Novocain) is an ester, and the patient is mostly likely to tolerate this medication without issue*.
- 7. A 42-year-old female with systemic lupus erythematosus (SLE), diabetes, and hypertension on lisinopril, hydroxychloroquine, metformin, insulin, and prednisone, presents to the emergency department with right sided groin pain. She is well appearing and afebrile with normal vital signs. Her pain is gradual in onset and worsening, worse with movement, and weightbearing. Hip and pelvis x-rays are normal on your interpretation. MRI is ordered to confirm the diagnosis. Which of her medications do you suspect to be responsible?
  - Hydroxychloroquine
  - Insulin
  - Lisinopril
  - Metformin
  - **Prednisone**
    *Question stem is describing avascular necrosis (AVN) of the hip. Avascular necrosis of the hip is associated with high dose steroids but can occur in SLE even in the absence of steroid use. Pathogenesis of steroid induced AVN is debated, but proposed mechanisms include alteration of circulating lipids causing microembolic phenomena; another proposed theory is increase in bone marrow adipocyte size and number leading to obstruction of venous outflow*.
- 8. A 42-year-old man presents to the emergency department with pain in the left forearm. He cut himself while working outside 2 weeks ago and superglued the wound at home. He now has worsening pain, erythema and swelling. X-ray imaging shows bony erosion. What is appropriate empiric therapy for this condition?
  - Vancomycin and cephalexin
  - piperacillin -tazobactam and gentamicin
  - **Vancomycin and ceftriaxone**
  - Levofloxacin and ampicillin
    *The question stem describes osteomyelitis, in this case from local spread. Another common etiology is from hematogenous spread. Strep, staph, and aerobic gram-negative rods are the most common organisms. Treatment requires vancomycin and third or fourth gen cephalosporin. If the patient is stable.****ideal therapy should be delayed until operative samples**** and cultures can be taken during surgical debridement which is the cornerstone of therapy*.
- 9. A 9-year-old boy presents to your western Pennsylvania emergency department for his second recurrent episode of left knee pain and swelling. He spends a lot of time outside and had multiple tick bites last summer. On exam he is well appearing but has significant pain, swelling, and limitation of range of motion in the right knee with palpable effusion. He has an age-appropriate ECG and no headache or nerve palsy. What is the recommended oral therapy for this patient’s condition?
  - **Doxycycline**
  - Ceftriaxone
  - Clarithromycin
  - Trimethoprim sulfamethoxazole
    *Question stem is describing monoarticular arthritis from Lyme disease infection. Treatment of choice is doxycycline, now recommended in younger age groups as well if course is 21 days or less. Other treatment options include amoxicillin or cefuroxime for erythema migrans stage. Ceftriaxone, penicillin, cefotaxime are parenteral options for refractory disease of Lyme meningitis. Doxycycline will also treat anaplasmosis which can occur as a co-infection*.
- 10. A 58-year-old man with cirrhosis and low back pain presents to the emergency department with altered mental status. He is placed on the cardiac monitor and found to be in a wide complex tachycardia. He then begins seizing. Which of his medications for back pain is he most likely suffering from toxicity of?
  - Acetaminophen
  - Diclofenac
  - Hydrocodone
  - **Cyclobenzaprine**
    *Cyclobenzaprine (Flexeril) has a TCA (tricyclic antidepressant) like structure and can cause sodium channel blockade with cardiac arrhythmias and seizures at toxic levels. It also has hepatic metabolism, and levels can be dangerously increased in cases of hepatic dysfunction*.
- 11. A 61-year-old female presents to the emergency department with sudden onset of severe right lower leg pain and inability to ambulate. When the calf is squeezed there is no observed plantar flexion of the ankle. On medical record review, you note she was seen at an urgent care center last week and treated for sinusitis. Based on her current presentation what medication do you think she was most likely given?
  - Amoxicillin-clavulanate
  - **Moxifloxacin**
  - Doxycycline
  - Fluticasone
  - Azithromycin
    *This Question stem describes an Achilles tendon rupture. Quinolones are not recommended as first line therapy for even true bacterial sinusitis, but have been used for this disease process. They are associated with tendinopathy. This is thought to occur in 3–4 per 100,000 people taking fluoroquinolones. It tends to happen early in treatment course with a median of 8 days. There are multiple posited mechanisms for this phenomenon: 1. inhibition of cell proliferation in tendon cells by down-regulation of cyclin B and cyclin-dependent kinase 1. 2. inhibition of the spread and migration of tenocytes. 3. ciprofloxacin enhances the enzymatic activity of matrix metalloproteinase causing degradation of type I collagen*
    *Tsai WC, Yang YM. Fluoroquinolone-associated tendinopathy. Chang Gung Med J. 2011;34(5):461–467*.
- 12. A 44-year-old man presents to the emergency department with acute non-traumatic low back pain. He has no neurologic deficits, no fever or history of intravenous drug use, and no history of malignancy. Which of the following medications has the strongest evidence basis for treating these symptoms?
  - Acetaminophen
  - **NSAID**
  - Opioid
  - Benzodiazepine
  - Non benzodiazepine muscle relaxant
    *Meta-analysis has shown modest pain improvement with NSAID for acute low back pain. A 2016 Cochrane review concluded no benefit in acetaminophen compared to placebo. Evidence regarding muscle relaxants is conflicting for efficacy in treating low back pain and would not be recommended as first line therapy. Evidence is similarly poor for opiates when added to NSAIDs that there is no significant improvement in outcomes. Opiates are not recommended as first line therapy for low back pain*.

### Pulmonary and Critical Care Block Answers

1. You are caring for a 27-year-old asthmatic patient, presenting for severe dyspnea. Despite multiple doses of nebulized albuterol, subcutaneous epinephrine, and Bipap, the patient continues to struggle to breathe and becomes somnolent; you elect to proceed with rapid sequence intubation (RSI). Which of the following agents may provide additional benefits in addition to facilitating RSI?
  - Etomidate
  - Propofol
  - **Ketamine**
  - Fentanyl
    *Ketamine has bronchodilatory effects; the exact mechanism is unclear but theorized that it is related to inhibition of catecholamine reuptake, immunomodulation, and/or anticholinergic effects on vascular smooth muscle*.
2. A 79-year-old female presents to your emergency department with fever and altered mental status. Her initial vital signs are T 39.4°C, RR 27 HR 145 BP 65/30. You activate the sepsis power plan; however, despite 30 cc/kg of lactated Ringer’s (LR) her BP remains low 72/38, and she continues to decompensate and is obtunded. You believe she needs intubation. Which of the following induction agents is most likely to exacerbate her existing hypotension?
  - **Propofol**
  - Midazolam
  - Etomidate
  - Ketamine
    *Propofol works through GABA A agonism. It is unrelated to barbiturates or benzodiazepines. Hypotension likely mediated through a combination of vasodilation and sympatholysis*.
3. A 66-year-old male presents to your emergency department as a level 1 stroke alert for sudden onset of right-sided weakness 45 minutes ago. On arrival he is completely obtunded and you prepare for intubation. You would like to use succinylcholine to quickly regain a reliable neurologic exam. Which of the following is not a contraindication to use of succinylcholine?
  - History of myotonic dystrophy
  - **His acute stroke**
  - A history of 20% TBSA (total body surface area) burns 3 weeks ago
  - History of lower extremity paraplegia from spinal cord injury
  - History of malignant hyperthermia
    *Succinylcholine can cause dangerous hyperkalemia in patients with history of neuromuscular disease, chronic paralysis or large body surface area burns older than 72 hours. While a chronic hemiplegia or other paralysis from old stroke may be a contraindication for succinylcholine, an acute stroke is not*.
4. A 45-year-old man presents to the emergency department for facial swelling. He reports this has been going on for two days and has happened 6 or 7 times before and no one knows why. His only medical history is hypertension on amlodipine. He also reports he has had intermittent cramping abdominal pain over the past 2 years. His family history is notable for a sudden death in his mother from “her throat swelling up.” If he begins to have worsening swelling and respiratory compromise, what medication is most likely to treat the underlying pathophysiological mechanism?
  - Idarucizumab
  - **C1 inhibitor**
  - Methylprednisolone
  - Epinephrine
    *The stem describes hereditary angioedema. Caused by dysfunction or deficiency of C1 inhibitor. Bradykinin mediated angioedema. Treatment is to replace the C1 inhibitor. It does not respond to steroids or epinephrine. Icatibant is a bradykinin receptor antagonist, trade name Firazyr, and can be used for hereditary angioedema. It is dosed at 30 mg subQ every 6 hours*
    *Idarucizumab is an unrelated drug, reversal agent for dabigatran*.
5. A 2-year-old male presents to your emergency department for a cough. On exam the child is uncomfortable in appearance with a barking cough and audible stridor. He has no fever and is non-toxic in appearance. Lung sounds are clear aside from inspiratory stridor. Which of the following choices is the most appropriate regimen?
  - Nebulized epinephrine
  - **IM dexamethasone and nebulized epinephrine**
  - Nebulized budesonide and albuterol
  - Heliox
  - Nebulized albuterol
    *The stem is describing croup. Standard treatment for croup with stridor at rest will require both corticosteroid and racemic epinephrine. Nebulized steroids are not part of treatment. No role for bronchodilators when diagnosis is clear*.
6. A 3-year-old female presents to your freestanding emergency department for fever and respiratory distress. You walk in the room and find a lethargic appearing child, in a tripod position, drooling. Unfortunately, the child begins struggling more to breath and becomes somnolent. You are able to successfully intubate, and post intubation x ray shows the ET tube in appropriate position and a thumbprint sign. While making transfer arrangements, what would best medical management be?
  - Vancomycin and piperacillin-tazobactam
  - **Dexamethasone, vancomycin and ceftriaxone**
  - Clindamycin
  - Dexamethasone and doxycycline
    *Empiric broad spectrum treatment for epiglottitis, Steroids also recommended*.
7. A 19-year-old male presents to the emergency department for wheezing and shortness of breath. His vital signs are RR 45 HR 127 T 37.2°C BP 100/60. On exam he has diffuse inspiratory and expiratory wheeze with decreased air movement and O2 saturation of 92% You initiate therapy with continuous albuterol and ipratropium and dexamethasone. You consider adding IV magnesium. Meta-analyses have demonstrated which of the following patient-oriented outcomes?
  - Decreased need for intubation
  - Increased FEV1 (forced expiratory volume)
  - **Decreased hospital admission**
  - Decreased need for epinephrine
    *A 2014 Cochrane review found decreased hospital admission rates in patients receiving magnesium during asthma exacerbation. FEV1 is not a patient-centered outcome*
    *Kew KM, Kirtchuk L, Michell CI, Griffiths B. Intravenous magnesium sulfate for treating adults with acute asthma in the emergency department. Cochrane Database of Systematic Reviews 2014, Issue 1. Art. No.: CD010909. Accessed 02 January 2021. DOI: 10.1002/14651858.CD010909*
8. A 28-year-old man with no past medical history presents to the ED with chest pain and cough. Vital signs are T 38.7°C HR 93 RR 19 BP 120/84. On exam he has crackles in the right upper lung field but lungs are otherwise clear to auscultation. He is complaining of a cough with purulent sputum. He reports allergies to penicillin and cefuroxime. Your local antibiogram shows a high resistance rate of strep pneumoniae to azithromycin. What is appropriate antibiotic therapy?
  - Ciprofloxacin
  - **Doxycycline**
  - Vancomycin and levofloxacin
  - Clindamycin and moxifloxacin
    *The stem is describing community-acquired pneumonia (CAP). Doxycycline monotherapy is 1 of 3 recommended regimens for healthy adult per 2019 Infectious Diseases Society of America guidelines. Vancomycin not required routinely for CAP. Quinolones are not first line agents. In the truly penicillin allergic patients, an argument could be made for treatment with a respiratory fluoroquinolone, but not in the combinations above*.
9. A 75-year-old female presents to the emergency department for sudden onset of fever, chills, rigors, malaise and cough. Symptom onset was approximately 36 hours ago. Oseltamivir is prescribed for a presumed influenza infection. Which of the following has been a demonstrated benefit of this treatment?
  - Shorter time to defervescence
  - Decreased requirement for hospital admission
  - Decreased need for intubation
  - **Shortened duration of illness by approximately 16 hours**
    *Oseltamivir (Tamiflu) has not been shown to decrease time to defervescence, intubation, or hospitalization*.
10. A 29-year-old female presents to your emergency department for chest pain, leg swelling and dyspnea. Her past medical history is notable for type 1 diabetes, chronic kidney disease stage IV, Factor V Leiden deficiency. She is also 28 weeks pregnant. Bedside ultrasound demonstrates a lower extremity DVT (deep vein thrombosis). Empiric treatment with heparin is initiated. What medication is most likely to be her definitive anticoagulation therapy?
  - Enoxaparin
  - Rivaroxaban
  - Dabigatran
  - **Heparin**
    *Lovenox not a good choice for severe kidney disease. NOACS (novel oral anticoagulants) are not yet deemed safe in pregnancy*.
11. A 53-year-old Chinese immigrant presents to your emergency department for three weeks of productive cough, now with hemoptysis, as well as fever, malaise, night sweats and a 5 kg unintentional weight loss. Chest x-ray shows a cavitary lesion in the right upper lobe. What is appropriate antibiotic therapy for this patient?
  - Levofloxacin
  - Cefuroxime and azithromycin
  - Vancomycin and cefepime
  - **Rifampin, Isoniazid, pyrazinamide, and ethambutol (RIPE)**
    In the patient above which chronic disease state would likely require a change in initial therapy?
  - **Autoimmune hepatitis**
  - Type 1 diabetes mellitus
  - Polycystic kidney disease
  - Cerebral palsy
    *RIPE therapy is first line treatment for TB. Does have significant hepatotoxicity risk*.
12. A 3-year-old male presents to the emergency department in status epilepticus having already received one dose of lorazepam in the field by EMS. He is accompanied by his grandmother who is concerned that he may have gotten into some of her medicine she has been taking for the past several months for a lung infection. What treatment should you initiate?
  - **Pyridoxine**
  - Physostigmine
  - Sodium bicarbonate
  - Fosphenytoin
  - Levetiracetam
    *Seizure from isoniazid toxicity requires supplementation of pyridoxine (vitamin B6):*
  - -*INH (isonicotinic acid hydrazide) directly binds and inactivates pyridoxine*.
  - -*INH inhibits pyridoxine phosphokinase which is necessary for production of pyridoxine*
    *INH does also decrease GABA because it is produced through pyridoxine-dependent decarboxylation, and this patient should receive benzodiazepines as well*.
13. A 9-year-old female presents to the emergency department for 3 weeks of persistent cough. This was preceded by fever with dry cough and rhinorrhea. Parents report that coughing spells tend to come in bursts followed by a forceful inspiration. After making the correct diagnosis, you assess the need to provide prophylaxis for close contacts. Who would require prophylaxis and with what drug?
  - His 4-year-old brother and his father with doxycycline
  - His father with trimethoprim sulfamethoxazole (TMP/SMx)
  - Anyone in the ED waiting room with azithromycin
  - **The ED physician who is 37 weeks pregnant with azithromycin**
    *The stem describes pertussis. Only close contacts require prophylaxis (so not the entire waiting room). Azithromycin is the preferred agent. TMP/SMx is an acceptable alternative in macrolide allergic patients*.
14. A 45-year-old man with a history of asthma, obesity, coronary artery disease, hypertension, diabetes, and obesity presents to the ED with 28 hours of fevers, chills, malaise, cough and myalgias. You clinically suspect influenza and plan to initiate therapy with oseltamivir. Which physiologic parameter is most important to consider when writing the appropriate treatment regimen?
  - Weight
  - Age
  - Oxygen saturation
  - **Renal function**
  - Hepatic function
    *Oseltamivir requires renal dosing because it is renally cleared*.
15. A 37-year-old man with a history of cystic fibrosis (CF) presents to the emergency department for shortness of breath and worsening of chronic cough. He has a new 2L oxygen requirement and is febrile at 38.2°C. In addition to broad spectrum and anti-pseudomonal antibiotic coverage, which of the following pharmacologic interventions is most likely to be helpful?
  - Sildenafil
  - **Dornase alfa**
  - High flow non-humidified oxygen
  - Ipratropium bromide
    *Dornase alpha selectively cleaves DNA. Airway secretions in CF patients have large amounts of polymerized DNA from breakdown of nuclei of neutrophils*.
    *Sildenafil can be used in pulmonary hypertension, but not likely to help this patient. The patient only requires 2L O2 currently and does not require high-flow nasal canula at this time. There is no clear role for ipratropium because CF is not a reactive airway disease*.
16. A 68-year-old female with a history of DVT, daily tobacco use, and on hormone replacement therapy presents to the emergency department for chest pain. During her stay she becomes tachycardic, hypotensive and diaphoretic. Bedside ultrasound shows right ventricle dilation and septal bowing. You elect to give TPA (tissue plasminogen activator), what is the dosing in this situation?
  - 1 mg/hr over 24 hrs
  - 50 mg over 2 minutes followed by 50 mg over 15 minutes
  - **100mg over 2 hours**
  - 15 mg over 1–2 minutes then 50 mg over 30 min, then 35 mg over 1 hour
    *A=catheter-directed dose. B=pulmonary embolism causing cardiac arrest. D= STEMI dose*
    What would your answer be if the patient above suffered from cardiac arrest?
  - 1 mg/hr over 24 hrs
  - **50 mg over 2 minutes followed by 50 mg over 15 minutes**
  - 100mg over 2 hours
  - 15 mg over 1–2 minutes then 50 mg over 30 min, then 35 mg over 1 hour

### Trauma Block Answers

1. A 24-year-old male presents to the ED after diving into the shallow end of the pool. Initial vital signs are T 36.4°C HR 84 BP 72/50 O2 sat 97%. Patient is unable to move his arms or legs, but is alert and answers questions. FAST exam is negative. In addition to crystalloid infusion, and further evaluation for thoracoabdominal injuries, what medication should be administered at this time?
  - Vasopressin
  - Tranexamic acid
  - **Norepinephrine**
  - Dopamine
  - PCC
    *The question stem above describes a patient that is significantly hypotensive along with a relative bradycardia. Given the mechanism of injury, this is concerning for neurogenic shock*
    *Source debate whether nor-epi or phenylephrine is the first line agent for neurogenic shock. Classic teaching was phenylephrine; most now recommending levophed, citing reflex bradycardia with phenylephrine*.
2. An 84-year-old woman presents to the ED as a level 1 trauma activation after a rollover motor vehicle collision (MVC). On arrival she is hypotensive with a FAST exam positive for fluid in the left upper quadrant. The hospital’s only trauma surgeon is already in the operating room with another patient. In addition to balanced transfusion of blood products, what other agents may be indicated in the treatment of this patient’s condition?
  - **Tranexamic acid**
  - Calcium Gluconate
  - Albumin
  - Andexanet alfa
    *CRASH-2 randomized controlled trial. Lancet. 2011; 377:1096–1101 demonstrating benefit of TXA within 3 hours of injury*
    *Calcium may be required for supplementation in massive transfusion patients, but most likely lifesaving intervention from choices above is TXA. DOI:https://doi.org/10.1016/S0140-6736(11)60278-X*
3. A 33-year-old man presents to the ED following a 17 foot fall off of a rooftop. Evaluation reveals a fracture to the left calcaneus and you decided to perform a posterior tibial nerve block for analgesia. He reports a previous anaphylactic allergic reaction to lidocaine; which anesthetic may you safely use?
  - Bupivacaine
  - Ropivacaine
  - Mepivacaine
  - **Tetracaine**
    *Lidocaine is an amide class anesthetic; tetracaine is the only listed ester option, is safe to use in this patient. Although typically used topically, tetracaine does have an IV form*.
4. A 12-year-old girl with type I diabetes presents to the emergency department 3 hours after being bitten on the dorsum of her hand by the family cat. She has a Penicillin allergy. In addition to local wound care, what therapy would you initiate?
  - Ibuprofen
  - Amoxicillin-clavulanate
  - Metronidazole plus doxycycline
  - **Clindamycin plus trimethoprim-sulfamethoxazole**
    *First line for animal bites is usually augmentin. However, since this patient is allergic to penicillin, doxycycline or clindamycin + ciprofloxacin or Bactrim would be appropriate*.
5. A 42-year-old artisan was etching glassware when he spilled a large amount of concentrated acid on his hand. He is in severe pain and his EKG showed QT prolongation. Which agent is most important in his treatment?
  - **Calcium gluconate**
  - Sodium Bicarbonate
  - IV Normal Saline
  - Potassium Chloride
    *Hydrofluoric acid (HF) burns require calcium gluconate. Topical paste can be used for mild skin exposures, but more significant exposures (as seen by QT prolongation in this question) require intravenous or even intraarterial therapy. Hydrofluoric acid chelates calcium and as a result hypocalcemia can result. Hypomagnesemia may also result. Additionally, HF can cause hyperkalemia*.
6. A 22-year-old male presents to the emergency department after a motorcycle accident. After primary and secondary survey and CT imaging of the head, chest, abdomen, pelvis and cervical, thoracic and lumbar spines, his only injury is a Gustilo II open tibia fracture. What is appropriate therapy for this injury?
  - Levofloxacin
  - **Cefazolin**
  - Vancomycin
  - Piperacillin-tazobactam
    *Gustilo I and II grade open fx need only first-generation cephalosporin. If the patient is allergic to cephalosporins, clindamycin or vancomycin may be used. Grade III needs broader coverage (piperacillin-tazobactam) as do injuries associated with aquatic environments*.
7. A healthy 47-year-old male presents to your emergency department with 7% TBSA (total body surface area) deep partial thickness burns. Which of the following topical dressings or treatments is relatively contraindicated in this patient due to a possible delay in healing?
  - Bacitracin and xeroform
  - Mepilex
  - **Silvadene**
  - Mupirocin and xeroform
    *Silvadene inhibits keratinocytes and should not be used for partial thickness burns*
    *Although this is usually the antibiotic of choice in third-degree burns, it inhibits keratinocyte replication which is problematic for second-degree burns because it inhibits healing. Silver sulfadiazine has been shown to inhibit healing and increase scarring of second-degree burns*.
8. A 62-year-old diabetic male presents to the emergency department today after finding a nail in his shoe when he took it off today. He has minimal sensation in the bottom of his feet due to neuropathy and is unsure when he may have stepped on this nail. Exam reveals a puncture wound on the plantar surface of the foot and a 1.5 cm area of surrounding erythema. What is appropriate therapy to initiate?
  - **Piperacillin tazobactam**
  - Vancomycin
  - Doxycycline
  - Cefazolin
    *Pseudomonal coverage for puncture wound through footwear, especially rubber-soled shoes, and therefore piperacillin tazobactam is the correct choice because the other antibiotics do not offer adequate pseudomonal coverage*.
9. A 101-year-old female presents to the emergency department with headache and confusion after a ground level fall. Her medications include diltiazem and rivaroxaban for atrial fibrillation. Head CT shows left-sided parietal subdural hematoma w/2 cm of midline shift. Which of the following therapies is most appropriate?
  - Vitamin K IV
  - FFP
  - Protamine Sulfate
  - **Prothrombin Complex Concentrate**
    *This patient is suffering life-threatening hemorrhage on rivaroxaban and needs immediate reversal of her anticoagulation along with emergent neurosurgical consultation. Andexanet alfa is a factor Xa inhibitor antidote but is very expensive and may not be readily available. If Andexanet alfa is not available, prothrombin complex concentrate (PCC) should be given*.
10. A 24-year-old previously unvaccinated man presents to the emergency department with a deep thigh laceration from a pitchfork used daily in the barn for his animals. What is the appropriate tetanus prophylaxis for this patient?
  - Tdap
  - Td
  - Tetanus immune globulin (Tig)
  - **Tdap + Tetanus immune globulin**
    *Dirty or wounds at high risk for tetanus in patients with less than 3 vaccinations need Tetanus immune globulin (TIg) for post exposure prophylaxis in addition to Tdap*.
